# Negative correlation between rates of molecular evolution and flowering cycles in temperate woody bamboos revealed by plastid phylogenomics

**DOI:** 10.1186/s12870-017-1199-8

**Published:** 2017-12-21

**Authors:** Peng-Fei Ma, Maria S. Vorontsova, Olinirina Prisca Nanjarisoa, Jacqueline Razanatsoa, Zhen-Hua Guo, Thomas Haevermans, De-Zhu Li

**Affiliations:** 1 Plant Germplasm and Genomics Center, Germplasm Bank of Wild Species, Kunming Institute of Botany, Chinese Academy of Sciences, Kunming, Yunnan 650201 China; 2 Comparative Plant and Fungal Biology, Royal Botanic Gardens, Kew, Richmond, Surrey TW9 3AB UK; 3 Kew Madagascar Conservation Centre, Lot IIJ, 131B, Ambodivoanjo, Ivandry, Antananarivo 101, Madagascar; 4 Herbier, Département Flore, Parc Botanique et Zoologique de Tsimbazaza, BP 4096, Antananarivo 101, Madagascar; 5 Institut de Systématique, Évolution, Biodiversité (ISYEB) UMR 7205 Centre national de la recherche scientifique - Muséum national d’histoire naturelle - École Pratique des Hautes Études – Université Pierre et Marie Curie, Sorbonne Universités, CP39, 57 rue Cuvier, 75231 Paris Cedex 05, France

**Keywords:** Temperate woody bamboos, Infrequent flowering, Plastid phylogenomics, Molecular evolution, Rate heterogeneity, Generation time

## Abstract

**Background:**

Heterogeneous rates of molecular evolution are universal across the tree of life, posing challenges for phylogenetic inference. The temperate woody bamboos (tribe Arundinarieae, Poaceae) are noted for their extremely slow molecular evolutionary rates, supposedly caused by their mysterious monocarpic reproduction. However, the correlation between substitution rates and flowering cycles has not been formally tested.

**Results:**

Here we present 15 newly sequenced plastid genomes of temperate woody bamboos, including the first genomes ever sequenced from Madagascar representatives. A data matrix of 46 plastid genomes representing all 12 lineages of Arundinarieae was assembled for phylogenetic and molecular evolutionary analyses. We conducted phylogenetic analyses using different sequences (e.g., coding and noncoding) combined with different data partitioning schemes, revealing conflicting relationships involving internodes among several lineages. A great difference in branch lengths were observed among the major lineages, and topological inconsistency could be attributed to long-branch attraction (LBA). Using clock model-fitting by maximum likelihood and Bayesian approaches, we furthermore demonstrated extensive rate variation among these major lineages. Rate accelerations mainly occurred for the isolated lineages with limited species diversification, totaling 11 rate shifts during the tribe’s evolution. Using linear regression analysis, we found a negative correlation between rates of molecular evolution and flowering cycles for Arundinarieae, notwithstanding that the correlation maybe insignificant when taking the phylogenetic structure into account.

**Conclusions:**

Using the temperate woody bamboos as an example, we found further evidence that rate heterogeneity is universal in plants, suggesting that this will pose a challenge for phylogenetic reconstruction of bamboos. The bamboos with longer flowering cycles tend to evolve more slowly than those with shorter flowering cycles, in accordance with a putative generation time effect.

**Electronic supplementary material:**

The online version of this article (10.1186/s12870-017-1199-8) contains supplementary material, which is available to authorized users.

## Background

Molecular evolutionary rate heterogeneity among lineages, whereby some lineages experience significantly different rates compared to their close relatives, is widespread across the tree of life. This phenomenon has been well documented [1, 2] in various plant groups, such as vittarioid ferns [3], the eudicot order Caryophyllales [4], and commelinid monocots [5, 6]. The lineage-specific rate heterogeneity can become problematic in inferring and dating evolutionary events [7–9], leading to biases through violations of the model assumptions of sequence evolution. The homoplastic characters in sequences with high rates of evolution may result in the clustering of them irrespective of their true phylogenetic relationships, a problem known as long-branch attraction (LBA) [7, 10, 11].

Most studies addressing molecular evolutionary rate heterogeneity have attempted to identify a correlation between the rate and life history traits of the organism in question [12, 13]. Relationships with numerous traits such as body size, metabolic rate, growth form or generation time have been suggested [1, 2, 12, 14], but no consensus has so far been reached regarding which trait best explains the rate variations, particularly for plants [15]. Life history traits can co-vary significantly in plants and variation within a trait (e.g., annual versus perennial habit) often reflects differences in generation time [1]. It has been suggested that organisms with long generation times are evolving slower because their genomes are copied less frequently leading to fewer DNA replication errors accumulating per unit of time, a phenomenon referred to as the generation time effect [12, 16].

A classical case of such a lineage with slow molecular evolutionary rates is the woody bamboos (Poaceae, subfamily Bambusoideae) [17]. They are unusual in the generally rapidly evolving grass family [5, 6], differing by their woody habits and extremely infrequent flowering with intervals as long as 10 to 60 years in the majority of species [18, 19], and by the fact that flowering events have remained unrecorded for a number of these bamboos until recently [19, 20]. Exhibiting the phenomenon of mass flowering, the majority of woody bamboos are monocarpically reproducing clones with prolonged vegetative phases [18]. Numerous hypotheses have been proposed to explain the evolution of this semelparous mass flowering in woody bamboos, such as predator satiation [18] and bamboo fire cycle [21]. However, neither has been supported by sufficient evidence. The flowering of woody bamboos remains an enigma in the evolution of plants.

The slow molecular evolutionary rate of woody bamboos has already been documented for a long time [17], particularly in the temperate woody bamboos (tribe Arundinarieae) [22–24], a fact presumably linked with their long flowering cycles (i.e., generation time). In addition, significant substitution rate variation within these bamboos was unexpectedly demonstrated in a recent plastid phylogenomic analysis [25], despite an overall slow rate. Even though it seems intuitive, the hypothesis of a negative correlation between rates of molecular evolution and flowering cycles in the woody bamboos has not yet been rigorously tested based on empirical data. An obvious explanation is the lack of reliably documented flowering cycles for them, as the flowering episodes remain unpredictable and the intervals are often longer than a researcher’s career or even lifespan [18, 26]. It is also likely due to the fact that the overall plastid genome substitution rates are usually too few to be reliably estimated using the set of common molecular markers usually sequenced for these bamboos.

The Arundinarieae are economically and ecologically important bamboos, consisting of ~533 species with a broad range of morphological diversity [19, 20, 27]. They are distributed primarily in montane forests from East Asia to Sri Lanka and the southern tip of India, Madagascar, Africa mainland, and eastern North America, exhibiting a highly disjunct and asymmetric distribution with more than 95% of the species level diversity confined to East Asia [19, 20, 27]. The taxonomy of Arundinarieae is complex with numerous unresolved controversies on the definition of the genera and species [23, 24, 27]. The lack of informative molecular markers due to slow rates of molecular evolution have also posed a major challenge for the systematics of Arundinarieae. Earlier phylogenetic studies based on multiple plastid loci divided the tribe into 12 lineages (seven of which contain only one species), but the relationships among these remained unresolved [23, 24, 28, 29]. These relationships were largely established using plastid phylogenomics [25], though the position and affinities of two lineages with only one species from Africa remained enigmatic. The partial plastid genomes available from three of the lineages (annotated as I and II from Africa and X from East Asia in [25]) may be partly responsible for this lack of resolution. During our preparation of this paper, another study of Arundinarieae employing plastid phylogenomics was published with 9 newly sequenced nearly complete to complete genomes comprising those of the two controversial continental African taxa [30]. Nevertheless, the controversy remains and further work is needed. Before this study, representative species covering the whole extant distribution of the Arundinarieae were sampled in the complete plastid genome dataset [25, 30], with a single notable exception: the endemic Arundinarieae radiation in Madagascar. These isolated Malagasy bamboos are the least studied within the Arundinarieae, and only two species with a couple of plastid DNA loci were included in molecular analyses before the present study [23, 31] out of a total six recognized taxa [32].

In order to gain an understanding of contrasting rates of molecular evolution and flowering cycles in Arundinarieae, it was necessary to significantly increase the number of plastomes available for this group, especially by including the Malagasy lineages about which little was known regarding phylogenetic positions or molecular evolutionary rates [31]. In designing this study, we planned to combine the 27 published complete plastid genomes of temperate woody bamboos [25, 33], aiming to resolve key nodes in the Arundinarieae tree. We sequenced 15 new complete plastid genomes to build the plastid genomic dataset used here to assess the degree of molecular rate heterogeneity in the tribe and test for any association between the rates of molecular evolution and the flowering cycles of temperate woody bamboos.

## Results

### Plastid genomes in Arundinarieae and alignments

**Fig. 1:**
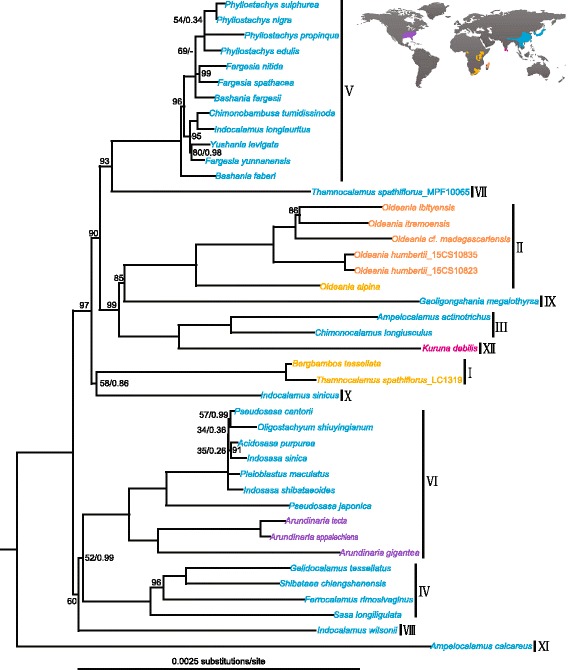
Phylogenetic relationships among major lineages of Arundinarieae based on complete plastid genomes. Unpartitioned maximum likelihood (ML) topology shown, and all unlabeled nodes received both maximum ML and Bayesian support values. The tropical woody bamboos as outgroups are not shown and the full tree is provided in Additional file 2

Using Illumina sequencing of total genomic DNA and a combination of de novo and reference-guide assembly, we were able to successfully assemble 15 new plastid genomes of temperate woody bamboos into a circular map without gaps. Among them, the plastid genomes of three lineages which were incomplete in a previous study [25] were re-sequenced, and plastomes from five individuals representing four Arundinarieae species native to Madagascar (Table 1) were sequenced for the first time. The 12 identified lineages of the tribe are now all represented in our dataset with complete plastid genome sequences (Table 1), also representing the full extent of the tribe’s distribution range (Fig. 1).

**Table 1:** List of the 40 Arundinarieae species sampled in this study, with flowering cycle data

Taxon	Lineage	Flowering period (Years)	Reference
***Bergbambos tessellata***	I	~45	[67]
***Oldeania alpina***	II	40	[68]
***Oldeania humbertii***	II	+	[19]
***Oldeania ibityensis***	II	+	[19]
***Oldeania itremoensis***	II	+	Field observation
***Oldeania*** **cf.** ***madagascariensis***	II	+	[19]
***Ampelocalamus actinotrichus***	III	+	[20]
*Chimonocalamus longiusculus*	III	+	[20]
*Ferrocalamus rimosivaginus*	IV	–	[20]
*Gelidocalamus tessellatus*	IV	+	[20]
***Sasa longiligulata***	IV	–	[20]
***Shibataea chiangshanensis***	IV	–	[20]
*Bashania faberi*	V	50–60	[69]
*Bashania fargesii*	V	70–75	[70]
***Chimonobambusa tumidissinoda***	V	+	[20]
*Fargesia nitida*	V	109 ~100	[71] [19]
*Fargesia spathacea*	V	+	[20]
*Fargesia yunnanensis*	V	+	[20]
*Indocalamus longiauritus*	V	+	[20]
*Phyllostachys edulis*	V	67	[72] [73]
*Phyllostachys nigra*	V	59–63	[18]
*Phyllostachys propinqua*	V	–	[20]
*Phyllostachys sulphurea*	V	>60 or 100	[18]
*Yushania levigata*	V	+	[20]
*Acidosasa purpurea*	VI	+	[20]
*Arundinaria appalachiana*	VI	+	[74]
*Arundinaria gigantea*	VI	20–25	[74]
*Arundinaria tecta*	VI	4–7?	[74]
***Indosasa shibataeoides***	VI	+	[20]
*Indosasa sinica*	VI	+	[20]
*Oligostachyum shiuyingianum*	VI	+	[20]
*Pleioblastus maculatus*	VI	+	[20]
***Pseudosasa cantorii***	VI	+	[20]
*Pseudosasa japonica*	VI	19	[75]
*Thamnocalamus spathiflorus*	VII	10	[18]
*Indocalamus wilsonii*	VII	+	[20]
*Gaoligongshania megalothyrsa*	IX	78	[76]
***Indocalamus sinicus***	X	+	[20]
*Ampelocalamus calcareus*	XI	+	Field observation
***Kuruna debilis***	XII	1 ≤5	[77] [78]

NOTE.—‘+’ denotes species with flowering recorded but without a reliably documented flowering cycle. ‘**-**’ denotes the species where flowering has never been recorded. Taxa in bold were newly sequenced in this study

Total genome sizes of the 15 new plastomes are similar, from 139,130 to 140,047 bp. Curiously, both the smallest (*Oldeania ibityensis*) and largest (*O. humbertii*) plastome have been newly sequenced from Madagascar (Additional file 1). No protein coding changes or significant genome structure variation such as gene order rearrangements or large inversions were detected in these newly generated plastid genomes. Four tropical woody bamboos (tribe Bambuseae) were chosen as outgroups for phylogenetic inference (Additional file 1), as these are identified as being the closest relatives to our group of interest [33]. For the ingroup, a total of 42 accessions representing 40 species in all the 12 lineages of Arundinarieae were sampled (Additional file 1). Multiple species were included from the three largest lineages (IV, V, and VI) to cover their phylogenetic diversity [23, 24]. We analyzed the whole plastid genome and the aligned matrix excluding one inverted repeat (IR) region was 124,679 bp in length. The sequence divergence was low at 3.03% across the Arundinarieae.

### Phylogenetic framework of Arundinarieae based on whole plastid genomes

In spite of the low sequence divergence, the complete plastid genomes allowed us to build a highly informative phylogenetic data set. We performed maximum likelihood (ML) and Bayesian analyses in both unpartitioned and partitioned ways. In the unpartitioned analysis, ML and Bayesian methods yielded identical tree topologies except for one node within lineage V (Fig. 1 and Additional file 2), but the support was poor in ML analyses (71% ML bootstrap value) and almost negligible in Bayesian analyses (0.34 Bayesian posterior probability). The optimal partition scheme selected by PartitionFinder [34] divided the alignment into five partitions, and nearly identical results were obtained for both the ML and Bayesian analyses (Additional file 3).

Overall, the phylogenetic relationships among the major lineages of Arundinarieae were essentially in agreement with those of [25, 30], but with better support especially for the nodes subtending lineages I, II and X. Seven of the ten nodes among the major lineages in our tree were strongly supported by ML bootstrap values ≥85% and Bayesian 1.0 posterior probabilities (Fig. 1). All the five sampled Malagasy bamboos form a monophyletic group, resolved with full support as sister to the genus *Oldeania* from eastern central Africa (Fig. 1). The sister relationship between lineages I and X, which contrasts with those of [25, 30], receives weak to moderate statistical support (58% ML bootstrap value and 0.86 Bayesian posterior probability). In addition, the ML bootstrap support values (52% and 60%) are still low for the relationships among lineages IV, VI and VIII [25, 30]. The two samples of *Thamnocalamus* from previous different studies [25, 33] were not clustered together (Fig. 1), most likely to be caused by the identity of the *T. spathiflorus*_LC1319 accession. This sample was clustered within the *Bergbambos* group while the *T. spathiflorus*_MPF10056 accession within the *Thamnocalamus* group both with 100% bootstrap support based on ML analysis of more *Bergbambos* and *Thamnocalamus* samples from [23, 24] (Additional file 4). The inferred tree features a series of short internodes connecting long terminal branches (Fig. 1), with striking differences in branch lengths among major lineages indicating varying rates of molecular evolution.

### Conflicting relationships and contrasting branch lengths

**Fig. 2:**
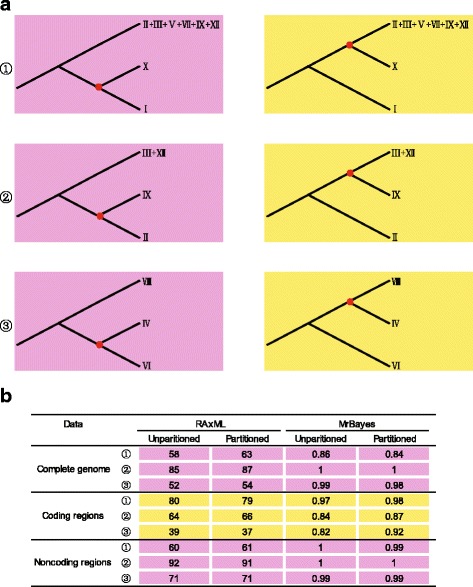
Summary of conflicting phylogenetic relationships involving the major lineages of Arundinarieae. The different topologies are illustrated in (**a**) and supports from reconstruction of different plastid genomic sequences and/or partitioning methods are summarized in (**b**)

Taking the potential effect of rate heterogeneity (e.g., LBA) into account, we analyzed the coding and noncoding sequences of the plastid genome in unpartitioned and partitioned schemes. These are different in their rates of molecular evolution which can be helpful to identify and overcome the potential systematic errors in our phylogenetic analyses. The phylogenetic relationships among the major lineages of Arundinarieae were largely consistent across analyses with either no partitioning or partitioning based on tRNA, rRNA, three codons of protein coding regions, or PartitionFinder delimited noncoding regions (Fig. 1 and Additional files 5, 6, 7 and 8). However, the sister group relationship between lineages II and IX was variable in addition to those moderately supported relationships mentioned above (Fig. 1) among different analyses. There are a total of three regions with conflict, enriched for short internodes and long branches in the phylogenetic tree, as summarized in Fig. 2.

Lineage X was recovered as sister to the monophyletic group comprising six major lineages in the coding dataset analyses, rather than to lineage I in all other analyses (Fig. 2). The highest ML bootstrap support value for the placement of lineage X was obtained in analyses of the coding dataset (80% in unpartitioned and 79% in partitioned; Fig. 2). On the other hand, the analyses of the coding dataset placed lineage IX as sister to lineages III + XII with moderate support rather than to lineage II (Fig. 2). For the relationships among lineages IV, VI and VIII, alternative topologies were again obtained in the coding dataset analyses compared to all other analyses. None of the recovered relationships among lineages IV, VI and VIII were strongly supported (Fig. 2). In contrast to the coding dataset, the phylogenetic relationships inferred from the noncoding dataset were in full agreement with those from analyses of whole plastid genomes, and the support values were generally higher (Additional files 7 and 8).

### Rate heterogeneity in Arundinarieae

The phylogram estimated from ML and Bayesian analyses (Fig. 1 and Additional files 2 and 3) based on the whole plastid genome dataset displays highly heterogeneous branch lengths for the major lineages of the Arundinarieae. There is a clear trend toward longer branches in the clade comprising lineages II, III, IX and XII while the shortest branches within Arundinarieae are found in lineage V (Fig. 1). Substantial branch length variation was also observed within lineage VI (Fig. 1). In addition to a simple visual inspection of branches lengths, we used the model-fitting test [3] provided by PAML [35] to formally investigate molecular evolutionary rate variation across the Arundinarieae.

The global clock model assuming equal rate among lineages was significantly rejected and a clockless model was mostly favored (Table 2), despite the strong penalty by the Akaike Information Criterion (AICc). The local clock models fitted the data better than the global clock model, but not as well as the clockless model. For the local clock models, the more lineages being allowed to have its own clock, the better fit of the model as evaluated by the AICc (Table 2). Each local clock model had an improvement of more than 12 points in AICc score over the next one with less numbers of lineages given their own clocks (Table 2) and four or more points could be considered significantly fitted [36]. Among them, the improvement was noteworthy by 125.698 AICc points in the model that allowed the grouping of the II, III, IX, and XII lineages to have its own clock, as expected given the extremely long branches for these lineages (Fig. 1). In addition, the largest variation in rate of molecular evolution within the tribe between the fastest- and slowest-evolving lineages is approximately a factor of three (XII and V) (Table 2).

**Table 2:** Fit of the nucleotide models using the baseml module of PAML for the complete plastid genome sequences

Model	Parameter count	lnL	AICc	Lineage rate (relative to background rate of 1)
Global Clock	54	−231,403.731	462,915.530	NA
Local Clock				
XI	55	−231,356.314	462,822.678	XI = 1.78149
XI, V	56	−231,330.487	462,773.025	XI = 1.73697 V = 0.53942
XI, V, (II + III + IX + XII)	57	−231,266.637	462,647.327	XI = 1.96045 V = 0.64983 II + III + IX + XII = 1.62495
XI, V, (II + III + IX + XII), VII, (I + X)	59	−231,258.371	462,634.799	XI =2.05983 V = 0.74772 II + III + IX + XII = 1.82538 VII =1.41126 I + X = 1.18578
Clockless	98	−229,978.490	460,153.136	NA

NOTE.—NA, not applicable in the listed analyses

**Fig. 3:**
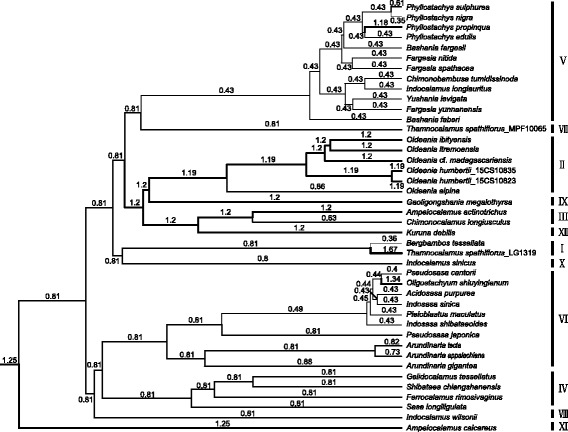
Relative plastid rate of molecular evolution among the lineages of Arundinarieae from Bayesian analysis under the random local clock (RLC) model. Branch thickness is proportional to the inferred median rate for the branch with detail numbers. The tropical woody bamboos as outgroups are not shown and the full tree is provided in Additional file 9

The BEAST analyses under the random local clock (RLC) model [37, 38] demonstrated consistent rate variation similar to the likelihood analyses, reconstructing molecular evolutionary rate for each branch of the Arundinarieae phylogenetic tree (Fig. 3 and Additional file 9). The number of rate shifts across the tree had a median value of 11 with 95% highest posterior density (HPD) interval of 9–13. The branches of lineages II, III, IX and XII were reconstructed as having increasing rates of molecular evolution while lineage V rates were decreasing, with a difference of nearly a factor of three between the two cases (Fig. 3). Rate decelerations also occurred within lineage VI (Fig. 3). In addition to the RLC model, we also applied a lognormal uncorrelated relaxed clock (LURC) model in BEAST analyses and obtained a very similar result, albeit with a less homogenous patterns of rate variation reconstructed (Additional file 10). Under this model, the rate decrease for lineage V appears to be largely attributable to a single slow branch at the base of the lineage, while rather uniformly elevated rates are inferred for lineages II, III, IX and XII (Additional file 10).

### Negative correlation between substitution rates and flowering cycles

**Fig. 4:**
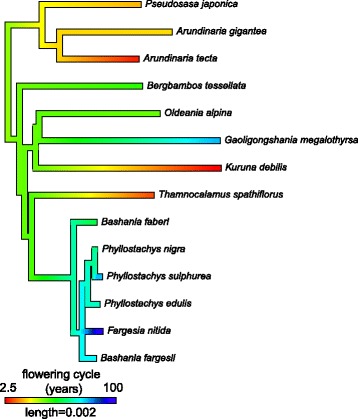
Reconstruction of the evolution of flowering cycles in Arundinarieae. The 14 documented records of flowering cycles are mapped on the pruned maximum likelihood (ML) tree in Fig. 1

As demonstrated above, it is now clear that extensive rate variation occurs across major lineages of the Arundinarieae in spite of the overall low rate of molecular evolution. As such, it is interesting to identify the potential correlates of such rate variation. A potential reason in bamboos would be the differences in the length of intervals between flowering events for which they are famous for, representing a sound measure of generation time. We first collected data on flowering events for the 40 Arundinarieae species sampled here from the literature. Fourteen of them have reliable records of flowering cycle, although the majority have data available for at least one documented flowering event (Table 1). To the best of our knowledge, these 14 records represent almost all the well-documented flowering cycles of Arundinarieae to date. The flowering cycles range from as short as 1 year to more than 100 years, with the majority between 20 to 60 years between each flowering event (Table 1). We also mapped the documented flowering cycles in the trimmed unpartitioned ML tree to demonstrate the evolution of this trait in the tribe (Fig. 4). The mapping reconstructed an average value of flowering cycle (indicated in green in Fig. 4) for the root of the tree with the very short or very long flowering cycles being derived. It is noted that *Gaoligongshania megalothyrsa* had a large value of flowering cycle length while a very long branch in the phylogenetic tree.

**Fig. 5:**
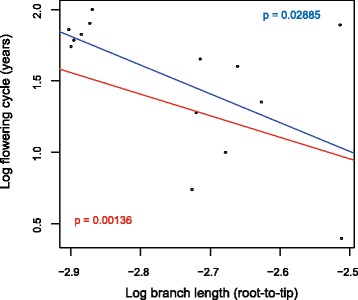
Rates of molecular evolution and flowering cycles are correlated in Arundinarieae. The flowering cycle is significantly negatively correlated with branch length inferred from unpartitioned maximum likelihood analysis using the simple (blue line) and Siegel’s repeated median (red line) linear regression. The filled circle represents the point of *Gaoligongshania megalothyrsa*

We calculated the root-to-tip branch lengths of the phylogenetic tree for each of these 14 species with documented flowering cycles, reflecting their rates of molecular evolution [13], and performed linear regression analysis to assess the relationship between rates and flowering cycles. Using the root-to-tip distances calculated on the ML tree inferred from the whole plastid genome sequences dataset (Fig. 1), a simple linear regression clearly showed a significant negative correlation between rates of molecular evolution and flowering cycles (*r* = −0.5824, *P* = 0.0288; Fig. 5). However, the point representing *G. megalothyrsa* is an outlier (the filled circle in Fig. 5), obviously deviating from the regression line. Therefore, we performed a linear regression using Siegel’s repeated median method which is insensitive to outliers [39], resulting in a more significant *p*-value of 0.00136 for the correlation (Fig. 5). In addition, we also performed a Phylogenetically Independent Contrasts (PIC) [39] analysis to account for phylogenetic relationships in detecting the correlation. The PIC analysis showed the same decreasing trend in rates of molecular evolution with flowering cycles, however, it was not significant (*r* = −0.1688, *P* = 0.5815).

## Discussion

With their idiosyncratic biology among the grasses (e.g., woody habit and infrequent flowering), the temperate woody bamboos have long been known for their extremely low rate of molecular evolution [17, 22–24]. The possible occurrence of rate heterogeneity within this group has been suggested [25]. However, systematic investigation of this hypothesis has been limited due to low genetic divergence, and limited availability of large-scale sequence data for phylogenetic reconstruction and substitution rate estimation [22–24]. Measuring rates of molecular evolution usually requires calculating branch lengths after phylogenetic reconstruction [13]. By generating complete plastid genome sequence data for a number of previously unsampled taxa, we have re-evaluated the phylogenetic relationships of Arundinarieae with all the 12 lineages sampled, providing further resolution for the phylogenetic placements of these isolated lineages within it. We also found strong evidence for extensive rate variation within temperate woody bamboos and tested the hypothesis that substitution rate is correlated with their flowering cycles.

### Substitution rate heterogeneity and correlates in Arundinarieae

Understanding molecular evolutionary rate variation between species presents challenges [3, 13]. Visual inspection of the differences among branch lengths of a phylogenetic tree can only give an empirical impression about the rate variation and rigorous statistical analyses should be used to test for the reality of the variation. We performed a likelihood-based model test on our dataset, a method shown to be an effective means of investigating rate heterogeneity in recent studies [3, 6], as well as a Bayesian framework to estimate relative substitution rates that can be compared directly. With the two approaches, we demonstrated extensive rate variation within the Arundinarieae (Fig. 3, Table 2). Although rate heterogeneity has been characterized for various groups of plants, the majority of studies have been carried out at high taxonomic levels, often above the family level [e.g., 3,4,6]. We present here an example of rate heterogeneity at a lower taxonomic level in plants, within a ca. 11–12 million years old tribe [31].

We found the rates of molecular evolution to vary substantially among major lineages of Arundinarieae, with isolated lineages showing a trend of elevated rates, especially the earliest diverging lineage XI and the grouping of lineages II, III, IX and XII (Fig. 3, Table 2). Rate decelerations mostly occur in lineage V which has the highest species diversity in the tribe [23, 24]. Lineage XI and the grouping of lineages II, III, IX and XII are evolving approximately three times faster, on average, than lineage V under various local clock models (Table 2), representing the greatest rate contrast within the Arundinarieae. A nearly identical range (2.79–2.91 times) of rate variation among these lineages was obtained in the Bayesian analyses (Fig. 3 and Additional files 9 and 10). In spite of at low taxonomic level, this largest rate differences bears some resemblance to those observed within high taxonomic levels such as the commelinid monocot (~3.53 times) [6] and the Caryophyllales (~3.1 times) [4]. The substitution rates of other isolated lineages are also high compared to lineage V, but not as dramatically as for those above. In addition to rate heterogeneity among major lineages, heterogeneity was also observed within lineage VI (Fig. 3 and Additional files 9 and 10). A total of 11 rate shifts during the evolution of Arundinarieae were recovered in the Bayesian analyses under the RLC model.

The slow rate of molecular evolution in woody bamboos has long been attributed to their infrequent flowering events [17]. Obtaining data on flowering cycles is a major challenge for study: historical recod of flowering events is poor due to their rarity and irregular nature [18, 26]. Existing historical records are usually fragmentary and verification is challenging, especially for the temperate woody bamboos [26]. With the 14 reliable records of flowering cycles collected in this study (Table 1) and linear regression analyses, we found a significant negative correlation between rates of molecular evolution and flowering cycles in Arundinarieae (Fig. 5), although the correlation was not significant when taking the shared evolutionary history of these bamboos into account with the PIC analysis. The nonsignificance of the PIC analysis may have been due to the small sample size for this test. Thus, the temperate woody bamboos evolving rapidly tend to have shorter flowering cycles than those evolving slowly, with *G. megalothyrsa* as a notable exception (Fig. 5). The mapping of flowering cycle on the tree indicates that the long flowering cycle of *G. megalothyrsa* may be a recent acquisition (Fig. 4). As expected, more significant *p*-values for the correlation were obtained using the Siegel’s repeated median linear regression method, which is a more powerful test for detecting a correlation in the presence of outliers and limited measurements [39], compared to the simple linear regression method (Fig. 5). Although the flowering cycles are not available for all the 40 sampled Arundinarieae species, these 14 flowering cycles are more or less evenly distributed in the major lineages of Arundinarieae (Figs. 1 and 4). As species from the same lineage have relatively consistent rates of molecular evolution and similar flowering cycle intervals (Figs. 3 and 4), we hypothesize that this negative correlation is likely to hold with the addition of flowering cycle data for more species. The flowering patterns and the factors triggering them in woody bamboos remain a mystery of biology [18, 21, 26]. The negative correlation with rate of molecular evolution established here for the first time is a formal test to be further explored for woody bamboos in future.

Generation time (as assessed by time between flowering cycle) effect would be a straightforward explanation for the correlation between rates of molecular evolution and flowering cycles in Arundinarieae [1, 12, 16]. It is generally expected that there would be more mutations accumulated with the increase in frequency of sexual reproduction per time unit due to short generation times. Although plant reproductive tissues develop from apical meristems that undergo continued mitosis, previous studies suggest that somatic mutations cannot counteract the influence of germline mutations on rates of molecular evolution [1]. Moreover, the generation time can also be used to explain the association between rates of molecular evolution and other traits, such as the height of the plants [40]. However, we cannot exclude the possibility that other biological process might be also involved in this correlation, especially for these isolated lineages with high rates of molecular evolution, species of which are usually endemic and narrowly distributed. Factors such as population size and bottleneck effects associated with past long-distance dispersal events in these bamboos [31] should be taken into account to explain the elevated rates in the isolated lineages, in particular those from Madagascar. Unfortunately, the synonymous and nonsynonymous substitutions of the plastid protein-coding genes which can provide more insights into understanding the potential roles of those factors [41, 42] are too low to be reliably estimated here. The negative correlation between rates of molecular evolution and flowering cycles as well as other potential correlates of rate variation across temperate woody bamboos warrant further investigation, incorporating other life history traits data [15], as well as nuclear and mitochondrial genomic data.

### Implications for the phylogeny and evolution of Arundinarieae

Rate heterogeneity can affect the accuracy of phylogenetic inferences [7–9], leading to the clustering of unrelated taxa with high molecular evolutionary rates, a well-described phenomenon known as LBA [10, 11]. The combination of molecular evolution model misspecification and highly heterogeneous rates can magnify this effect [9]. In the phylogenetic tree for the Arundinarieae, the relationships involving several isolated lineages (e.g., lineages IX and X) are difficult to resolve (Figs. 1 and 2) [25, 30]. Containing short-internodes combined with long terminal branches, they are prone to the influence of LBA. To tackle the potential systematic errors, we performed phylogenetic analyses using the coding sequences that are conserved in evolution as well as partitioned models. These approaches all have the ability to handle LBA artifacts [11, 43].

The sister relationship between lineages I and X was not supported in the analyses of the slowly evolving plastid coding sequences. Both the two lineages are rapidly evolving with long branch lengths (Fig. 1) and this sister relationship revealed by the analyses of the whole plastid genomes is likely to be a result of LBA. If this is the case here the effect of LBA would be enlarged in the analyses of the rapidly evolving plastid noncoding sequences [11] and indeed this is what we found (Fig. 2). For the sister relationship between lineages II and IX, we obtained a similar result as above (Fig. 2). The phylogenetic relationships involving lineages IV, VI and VIII — the remaining areas of uncertainty in the Arundinarieae tree — remain elusive (Fig. 2). Altogether, we suggest that phylogenetic relationships reconstructed using coding sequences are more reliable than those using noncoding or complete plastid genomic sequences, at least for the phylogenetic placements of lineages IX and X. Therefore, we suggest the plastid coding sequences should be analyzed independently from the complete plastid genomes despite the low molecular evolutionary rates in plant phylogenomics, even at lower taxonomic levels.

In addition to slow molecular evolutionary rate, rapid radiation, and reticulate evolution [23–25, 44], the high rate heterogeneity documented here as another major factor causing the poor resolution of Arundinarieae phylogeny, which has been neglected in previous studies. In spite of those challenges our phylogenomic study, with its unprecedented taxon sampling of whole plastid genomes devoid of missing data, still recovers a well-supported phylogeny of Arundinarieae. Overall, the phylogenetic relationships among the major lineages of the tribe are largely in accordance with earlier studies [25, 30, 31], but are better supported and provide novel insights into the evolution of Arundinarieae. The new phylogenetic placement of lineage VII (fide in [30]) did not represent a true conflict and likely arose from the issue of species misidentification (Additional file 4). One of the significant results is the full resolution of the phylogenetic placement of Malagasy temperate woody bamboos within the Arundinarieae, as sister to *Oldeania alpina* from eastern central Africa. This result is not unexpected because of the close geographical affinities and morphological similarities between them [45, 46]. This monophyletic group is nevertheless not sister to *Bergbambos*, another isolated lineage distributed in Africa, and the two lineages (I and II) have separate isolated lineages from East Asian as its closer relatives. Furthermore, the majority of these isolated lineages have a high rate of molecular evolution. It will be interesting to investigate the diversification and evolution of these isolated lineages, together with the “out of East Asia” hypothesis [31] given the generally low dispersal capability for these bamboos.

## Conclusions

Our analyses of whole plastid genomes further clarify the phylogenetic relationships among major lineages of Arundinarieae [25, 30], especially for the placements of these isolated lineages. Moreover, our study provide strong evidence for extreme heterogeneity of substitution rates within Arundinarieae and assess the correlation between rates of molecular evolution and flowering cycles for these bamboos. To our knowledge, this is the first example of test for this correlation. The bamboos with longer flowering cycles tend to evolve more slowly and we hypothesize that generation time effect can be the drivers of this correlation. The insights gained from our study have implications for the systematics of Arundinarieae and contribute to our general understanding of evolution of these bamboos.

## Methods

### Taxon sampling and data collection

Within the 46 bamboos sampled here, the complete plastid genomes of 31 bamboos were previously sequenced and downloaded from GenBank database, and the remaining 15 genomes were newly sequenced for this study (Additional file 1). We used leaf material for genome sequencing and the samples were collected in the field, including five samples from their native distributions in Madagascar. Research permits for collecting in Madagascar were issued by Madagascar National Parks and by Direction Générale des Forêts, facilitated by the Missouri Botanical Gardens and supported by Parc Botanique et Zoologique de Tsimbazaza and by the Kew Madagascar Conservation Centre, and no specific collecting permits required for the remaining samples. The voucher information for the newly sequenced bamboos is listed in S1 Table. We gathered information on flowering cycles for the 40 sampled Arundinarieae species by searching the relevant literature. When multiple published sources existed for a species, all the information was recorded. Unsurprisingly, the data on flowering cycle are not available for every of the sampled species and in the end only 14 species had data consistently collected (Table 1). The mean value of flowering cycle was taken where different records are present for a given species (2.5 years for *Kuruna debilis*) and all 14 records were mapped on the pruned ML tree (Fig. 1) using the contMap() function in the phytools package [47] to understand evolution of this trait in Arundinarieae.

### Plastid genome sequencing, assembly, and annotation

Total DNA was extracted from fresh or silica-dried leaf material using a modified CTAB protocol [48]. After quantifying with NanoDrop spectrophotometer (Thermo Scientific, Carlsbad, CA, USA) and gel electrophoresis, total DNAs were shipped to BGI-Shenzhen for library preparation and Illumina sequencing. Paired-end reads (90, 125 or 150 bp) were generated from a library with 500 bp insert size, totaling to 500 Mb to 2 Gb clean data after filtering for each sample with the exclusion of *O. alpina*. Genome survey sequencing of this species in another project produced approximately 30 Gb sequence data, and we only extracted a subset data of 1.5 Gb for subsequent plastid genome assembly.

De novo and reference-guided assembly was used in combination to build plastid genomes following [25]. Briefly, de novo assemblies were constructed in SOAPdenovo v1.05 [49], under k-mer values of 63–101 in different samples. The output scaffods/contigs larger than 1000 bp were retained and mapped with BLAST to the plastid genome of *Phyllostachys edulis* (GenBank HQ337796). The connection among mapped scffolds/contigs was then guided by the reference genome. The gaps in the assembly, if any, were closed by searching the corresponding reference sequence against all the assembled scaffods/contigs with BLAST or mapping reads with BWA v0.5.9 [50] and SAMtools v0.1.19 [51]. The Arundinarieae plastid genomes are conserved in evolution (e.g., gene content and order) [25, 30, 52], facilitating genome assembly and we obtained all the complete genomes. Subsequently, the assembled genomes were annotated using DOGMA [53]. We manually checked the boundaries of exons and introns as well as positions of start and stop codons with minor adjustments made if necessary. All the newly sequenced plastid genomes were deposited in GenBank database under the accession numbers MF066243-MF066257 (Additional file 1).

### Sequence alignment and data set construction

The complete plastid genome sequences for 46 bamboos excluding one of the IR regions were aligned with MAFFT v7.215 in the default settings [54]. The aligned sequences were then inspected manually in MEGA v6.0 [55], with limited ambiguous regions and identified small inversions removed. In addition, we adjusted the alignment of protein-coding sequences within the coding frame. To assess the effects of molecular evolutionary rate heterogeneity on phylogenetic estimation, we also analyzed two data subsets derived from the whole alignment: the combined coding (protein-coding genes, tRNAs, and rRNAs) and noncoding sequences, respectively. The coding sequences are presumed to be more conserved in evolution, while the noncoding sequences are more rapidly evolving. There are six protein-coding genes whose alignments are not in strict multiple of three in the end due to the mutations causing premature stop codons in some species, and to analyze the coding data partitioned by three codons (see below) we deleted these aligned sequences totaling 69 bp from these six genes. The final alignment was 62,831 bp and 61,779 bp for the coding and noncoding dataset, respectively.

### Phylogenetic analyses

We analyzed each of the three data matrices constructed above using ML and Bayesian methods in both unpartitioned and partitioned ways. PartitionFinder v1.1.1 [34] was used to find the optimal partition schemes for the datasets of whole alignment and noncoding, using the ‘rcluster’ algorithm and the Bayesian information criterion (BIC). For the whole alignment, the program was ran starting from 262 a priori defined partitions corresponding to the combined tRNAs, rRNAs, 80 noncoding regions (those < 200 bp were combined with nearby regions), and 3 codon positions by each of the 60 protein-coding genes (those < 200 bp were separately combined with genes from the same functional group and treated as one gene) following [25]. For the noncoding dataset, the priori defined partitions of 80 noncoding regions as above were used. On the other hand, we divided the coding dataset into 5 partitions corresponding to the combined tRNAs, rRNAs, and 3 codon positions of the concatenated protein-coding genes instead of using PartitionFinder.

ML inference was implemented with RAxML v8.0.20 [56] under the GTR + Γ model as suggested in the software manual. Analyses were conducted with 10 replicate ML searchers for the best-scoring ML tree, and 1000 bootstrap replicates were estimated using the standard bootstrap option. Bayesian inference was conducted using MrBayes v3.2.6 [57] under the best selected model of sequence evolution by jModelTest v 2.1.6 [58] for each dataset in the unpartitioned analyses (Additional file 11). In the partitioned analyses, the models determined by PartitionFinder for each identified optimal partition were used for the datasets of whole plastid genome and noncoding, while for the coding dataset the model was also selected by jModelTest for each of the five partitions defined above (Additional file 12). Two independent Markov Chain Monte Carlo (MCMC) chains were run, each with three heated and one cold chain starting with a random tree. MCMC was run for 2 or 4 (partitioned analysis of the whole plastid genome) million generations, with trees and model parameters being sampled every 100 generations. Discarding the first 25% of trees as burn-in after convergence checking, the remaining trees were used to build a 50% majority-rule consensus tree.

The two individuals of *T*. *spathiflorus* from different studies [25, 33] did not form a monophyletic group in our phylogenetic tree with *T. spathiflorus*_LC1319 as sister to *Bergbambos* (Fig. 1). This result is also noted in [30] and the possibility of misidentification was proposed as a potential reason. To further clarify this issue, all the *Thamnocalamus* and related *Bergbambos* samples in previous studies [23, 24], within which the major lineages of Arundinarieae were firstly defined, were added to our taxon sampling for phylogenetic analyses. ML inference was conducted as above using RAxML based on 7 plastid loci (*atpI*-*atpH*, *psaA*-*ORF170*, *rpl32*-*trnL*, *rps16*-*trnQ*, *trnC*-*rpoB*, *trnD*-*trnT*, and *trnT*-*trnL*) in these two studies.

### Plastid lineage-specific rate heterogeneity analyses

To explicitly assess variation in rate of molecular evolution across the Arundinarieae lineages, we adopted a model comparison approach using the baseml program of the PAML v4.8 package [35]. We first examined the global molecular clock model, assuming that all the bamboos are evolving at an identical molecular evolutionary rate. Afterwards, different local molecular clock models were tested, allowing rates to vary in predefined regions of the phylogenetic tree corresponding to the major Arundinarieae lineages. For the local clock model, four distinct regimes were chosen according to the observed variations among branch lengths of the major lineages in the tree (Fig. 1), which are of particular biological interest for comparison. All of our analyses utilized a GTR + Γ model and fixed topology obtained in unpartitioned analysis of the whole plastid genome (Fig. 1). Each analysis was repeated 10 times independently to avoid suboptimal peaks, and the highest likelihoods from the runs were reported here.

To further investigate the variation in rate of molecular evolution in detail, a Bayesian approach was taken for our whole plastid genome dataset using BEAST v1.8.0 [37, 38]. The dataset was analyzed using two relaxed clock models implemented in the BEAST software, the RLC and LURC models [59], respectively. In both cases, we employed an unpartitioned GTR + Γ + I model and a Yule tree prior. Monophyly was enforced for four taxon sets based on previous studies [23–25] and phylogenetic results here: all Arundinarieae taxa, and all taxa of three lineages IV, V, and VI, respectively. The priors were left at their default values. As we are not interested in the absolute divergence time estimation here, the fossil calibrations were not used and the average clock rate was fixed at 1.0. Convergence was assessed in Tracer v.1.6, with effective sample sizes (ESS) above 200 for the parameters. For the LURC model, the analysis was run 200 million generations three times independently, with each sampled every 2000 generations. These runs converged relatively rapidly and there was only one parameter with an ESS value of 196 under 200 in one of them. For the RLC model, seven times were run with 200 million generations sampling every 2000 generations, however, three individual runs failed to converge and were abandoned. The first 40 and 80 million generations in individual runs were discarded as burn-in for the LURC and RLC models, respectively, and the remaining trees were summarized using TreeAnnotator v1.8.0.

### Test of correlation between rates of molecular evolution and flowering cycles

To test for a relationship between rates of molecular evolution and flowering cycles, we calculated the root-to-tip branch lengths of the phylogenetic trees for the 14 tips of bamboo species with documented flowering cycles. As each bamboo tip in the tree has had the same amount of time to evolve since split from their shared common ancestor, any differences in the root-to-tip branch lengths can reflect a difference in rate of molecular evolution [13]. The trees were trimmed to keep only these 14 tips using the function drop.tip() in the R package ape [60] and the patristic root-to-tip distances were computed using the distRoot() function in the R package adephylo [61].

We first implied a simple linear regression method using the lm() function in R [62] on log-transformed flowering cycles and patristic distances calculated from unpartitioned ML tree based on the whole plastid genomes. As our dataset has only a limited amount of measurements compared to the complete tree we are analyzing and obvious outlier (the point of *G. megalothyrsa*, see details in Results), we also performed a linear regression using Siegel’s repeated median method [40] with the mblm() function in the mblm R package [63]. This more sophisticated method is a variant of Theil-Sen’s single median estimator [64, 65], which is documented as being robust for estimating the true slope in a simple linear regression and insensitive to outliers. Finally, we also used the PIC analysis [66], which was implemented in the R package ape [60] to overcome the potential problem of nonindependence as closely related species tend to be similar because of shared evolutionary history.

## Additional files

Additional file 1: List of 46 bamboo taxa sampled in this study with related voucher and GenBank accession information. (DOC 97 kb)

Additional file 2: Phylogenetic relationships among major lineages of Arundinarieae based on complete plastid genomes in unpartitioned maximum likelihood (ML) (A) and Bayesian analysis (B). Values associated with the nodes indicate the ML or Bayesian support values and the unlabeled nodes receive 100% ML bootstrap support or 1.0 Bayesian posterior probability. (PDF 389 kb)

Additional file 3: Phylogenetic relationships among major lineages of Arundinarieae based on complete plastid genomes in partitioned maximum likelihood (ML) (A) and Bayesian analysis (B). The partitioned scheme was determined by the software PartitionFinder. Values associated with the nodes indicate the ML or Bayesian support values and the unlabeled nodes receive 100% ML bootstrap support or 1.0 Bayesian posterior probability. (PDF 395 kb)

Additional file 4: Maximum likelihood (ML) phylogeny of Arundinarieae based on 7 plastid loci (*atpI-atpH*, *psaA-ORF170*, *rpl32-trnL*, *rps16-trnQ*, *trnC-rpoB*, *trnD-trnT*, and *trnT-trnL*) for the clarification of the phylogenetic placement of *Thamnocalamus spathiflorus* LC1319. These 7 plastid loci are from Triplett & Clark (2010), Zeng et al. (2010), Wysocki et al. (2015) (references in the main document), and our sampled complete plastid genomes. The DNA sequences of *T. spathiflorus* LC1319 are from Wysocki et al. (2015) and the *T. spathiflorus* MPF10056 is our sampling. All the remaining individuals of *T. spathiflorus* are from Zeng et al. (2010). Values associated with nodes indicate ML bootstrap support values, with only ≥70% shown. (PDF 353 kb)

Additional file 5: Phylogenetic relationships among major lineages of Arundinarieae based on plastid coding sequences in unpartitioned maximum likelihood (ML) (A) and Bayesian analysis (B). Values associated with the nodes indicate the ML or Bayesian support values and the unlabeled nodes receive 100% ML bootstrap support or 1.0 Bayesian posterior probability. (PDF 409 kb)

Additional file 6: Phylogenetic relationships among major lineages of Arundinarieae based on plastid coding sequences in partitioned maximum likelihood (ML) (A) and Bayesian analysis (B). Five partitions corresponding to the tRNA, rRNA, and three codon positions of protein-coding genes were selected. Values associated with the nodes indicate the ML or Bayesian support values and the unlabeled nodes receive 100% ML bootstrap support or 1.0 Bayesian posterior probability. (PDF 403 kb)

Additional file 7: Phylogenetic relationships among major lineages of Arundinarieae based on plastid noncoding sequences in unpartitioned maximum likelihood (ML) (A) and Bayesian analysis (B). Values associated with the nodes indicate the ML or Bayesian support values and the unlabeled nodes receive 100% ML bootstrap support or 1.0 Bayesian posterior probability. (PDF 399 kb)

Additional file 8: Phylogenetic relationships among major lineages of Arundinarieae based on plastid noncoding sequences in partitioned maximum likelihood (ML) (A) and Bayesian analysis (B). The partitioned scheme was determined by the software PartitionFinder. Values associated with the nodes indicate the ML or Bayesian support values and the unlabeled nodes receive 100% ML bootstrap support or 1.0 Bayesian posterior probability. (PDF 400 kb)

Additional file 9: Relative plastid rate of molecular evolution among the lineages of Arundinarieae from Bayesian analysis under the random local clock (RLC) model. Branch thickness is proportional to the inferred median rate for the branch with detail numbers. (PDF 365 kb)

Additional file 10: Relative plastid rate of molecular evolution among the lineages of Arundinarieae from Bayesian analysis under the lognormal uncorrelated relaxed clock (LURC) model. Branch thickness is proportional to the inferred median rate for the branch with detail numbers. (PDF 372 kb)

Additional file 11: Sequence length and model selected for the three data sets used in the study. (DOC 29 kb)

Additional file 12: Sequence length and model selected for each data partition of the coding data set. (DOC 31 kb)

## Abbreviations

AICc: Akaike information criterion
ESS: Effective sample size
HPD: highest posterior density
LBA: Long-branch attraction
LURC: Lognormal uncorrelated relaxed clock
MCMC: Markov Chain Monte Carlo
ML: Maximum likelihood
PIC: Phylogenetically independent contrasts
RLC: Random local clock
