# Supplementary material for: Negative correlation between rates of molecular evolution and flowering cycles in temperate woody bamboos revealed by plastid phylogenomics
Source: BMC Plant Biol. 2017 Dec 21;17:260. doi: 10.1186/s12870-017-1199-8 (PMC5740905; doi:10.1186/s12870-017-1199-8)
Supplement: Supplementary file 1 — List of 46 bamboo taxa sampled in this study with related voucher and GenBank accession information. (DOC 97 kb) [file 12870_2017_1199_MOESM1_ESM.doc]

Table S1. List of 46 bamboo taxa sampled in this study with related voucher and GenBank accession information.

| Taxon | Clade | Voucher specimen | Genome  size (bp) | Accession numbers |
| --- | --- | --- | --- | --- |
| Arundinarieae |  |  |  |  |
| ***Bergbambos tessellata*** | Ⅰ | KMBG1301 | **139,564** | MF066246 |
| ***Oldeania alpina*** | Ⅱ | DZL1506 | **139,599** | MF066243 |
| ***Oldeania humbertii*** | Ⅱ | 15CS10823 | **140,047** | MF066247 |
| ***Oldeania humbertii*** | Ⅱ | 15CS10835 | **140,046** | MF066248 |
| ***Oldeania ibityensis*** | Ⅱ | 15CS10809 | **139,130** | MF066249 |
| ***Oldeania itremoensis*** | Ⅱ | 15CS10806 | **139,751** | MF066252 |
| ***Oldeania* cf. *madagascariensis*** | Ⅱ | 15CS10839 | **139,649** | MF066254 |
| ***Ampelocalamus actinotrichus*** | Ⅲ | MPF10003 | **139,774** | MF066245 |
| *Chimonocalamus longiusculus* | Ⅲ | NA | 139,821 | NC_024714 |
| *Ferrocalamus rimosivaginus* | Ⅳ | NA | 139,467 | NC_015831 |
| *Gelidocalamus tessellatus* | Ⅳ | NA | 139,712 | NC_024719 |
| ***Sasa longiligulata*** | Ⅳ | GY15048A | **139,928** | MF066256 |
| ***Shibataea chiangshanensis*** | Ⅳ | ZLN-2011080 | **139,737** | MF066257 |
| *Bashania faberi* | Ⅴ | NA | 139,629 | NC_024713 |
| *Bashania fargesii* | Ⅴ | NA | 139,696 | NC_024712 |
| ***Chimonobambusa tumidinoda*** | Ⅴ | MPF10083 | **139,502** | MF066244 |
| *Fargesia nitida* | Ⅴ | NA | 139,535 | NC_024715 |
| *Fargesia spathacea* | Ⅴ | NA | 139,767 | NC_024716 |
| *Fargesia yunnanensis* | Ⅴ | NA | 139,609 | NC_024717 |
| *Indocalamus longiauritus* | Ⅴ | NA | 139,668 | NC_015803 |
| *Phyllostachys edulis* | Ⅴ | NA | 139,679 | NC_015817 |
| *Phyllostachys nigra* | Ⅴ | NA | 139,839 | NC_015826 |
| *Phyllostachys propinqua* | Ⅴ | NA | 139,704 | NC_016699 |
| *Phyllostachys sulphurea* | Ⅴ | NA | 139,731 | NC_024669 |
| *Yushania levigata* | Ⅴ | NA | 139,633 | NC_024725 |
| *Acidosasa purpurea* | Ⅵ | NA | 139,697 | NC_015820 |
| *Arundinaria appalachiana* | Ⅵ | NA | 139,547 | NC_023934 |
| *Arundinaria gigantea* | Ⅵ | NA | 138,935 | NC_020341 |
| *Arundinaria tecta* | Ⅵ | NA | 139,499 | NC_023935 |
| ***Indosasa shibataeoides*** | Ⅵ | MPF10028 | **139,711** | MF066251 |
| *Indosasa sinica* | Ⅵ | NA | 139,660 | NC_024721 |
| *Oligostachyum shiuyingianum* | Ⅵ | NA | 139,647 | NC_024722 |
| *Pleioblastus maculatus* | Ⅵ | NA | 139,720 | NC_024723 |
| ***Pseudosasa cantorii*** | Ⅵ | MPF10006 | **139,689** | MF066255 |
| *Pseudosasa japonica* | Ⅵ | NA | 139,641 | NC_028328 |
| *Thamnocalamus spathiflorus* | Ⅶ | NA | 139,778 | NC_024724 |
| *Thamnocalamus spathiflorus* | Ⅶ | NA | 139,498 | KJ871005 |
| *Indocalamus wilsonii* | Ⅷ | NA | 139,962 | NC_024720 |
| *Gaoligongshania megalothyrsa* | Ⅸ | NA | 140,064 | NC_024718 |
| ***Indocalamus sinicus*** | Ⅹ | ZMY037 | **139,619** | MF066250 |
| *Ampelocalamus calcareus* | Ⅺ | NA | 139,689 | NC_024731 |
| ***Kuruna debilis*** | Ⅻ | F1012116 | **139,971** | MF066253 |
| Outgroups |  |  |  |  |
| *Bambusa emeiensis* | NA | NA | 139,493 | NC_015830 |
| *Chusquea liebmannii* | NA | NA | 138,001 | NC_026969 |
| *Dendrocalamus latiflorus* | NA | NA | 139,350 | NC_013088 |
| *Guadua weberbaueri* | NA | NA | 135,324 | NC_026991 |

NOTE.—NA, not applicable. Taxon in bold was sequenced in this study.
