# Supplementary figures and images for: Negative correlation between rates of molecular evolution and flowering cycles in temperate woody bamboos revealed by plastid phylogenomics
Source: BMC Plant Biol. 2017 Dec 21;17:260. doi: 10.1186/s12870-017-1199-8 (PMC5740905; doi:10.1186/s12870-017-1199-8)

A

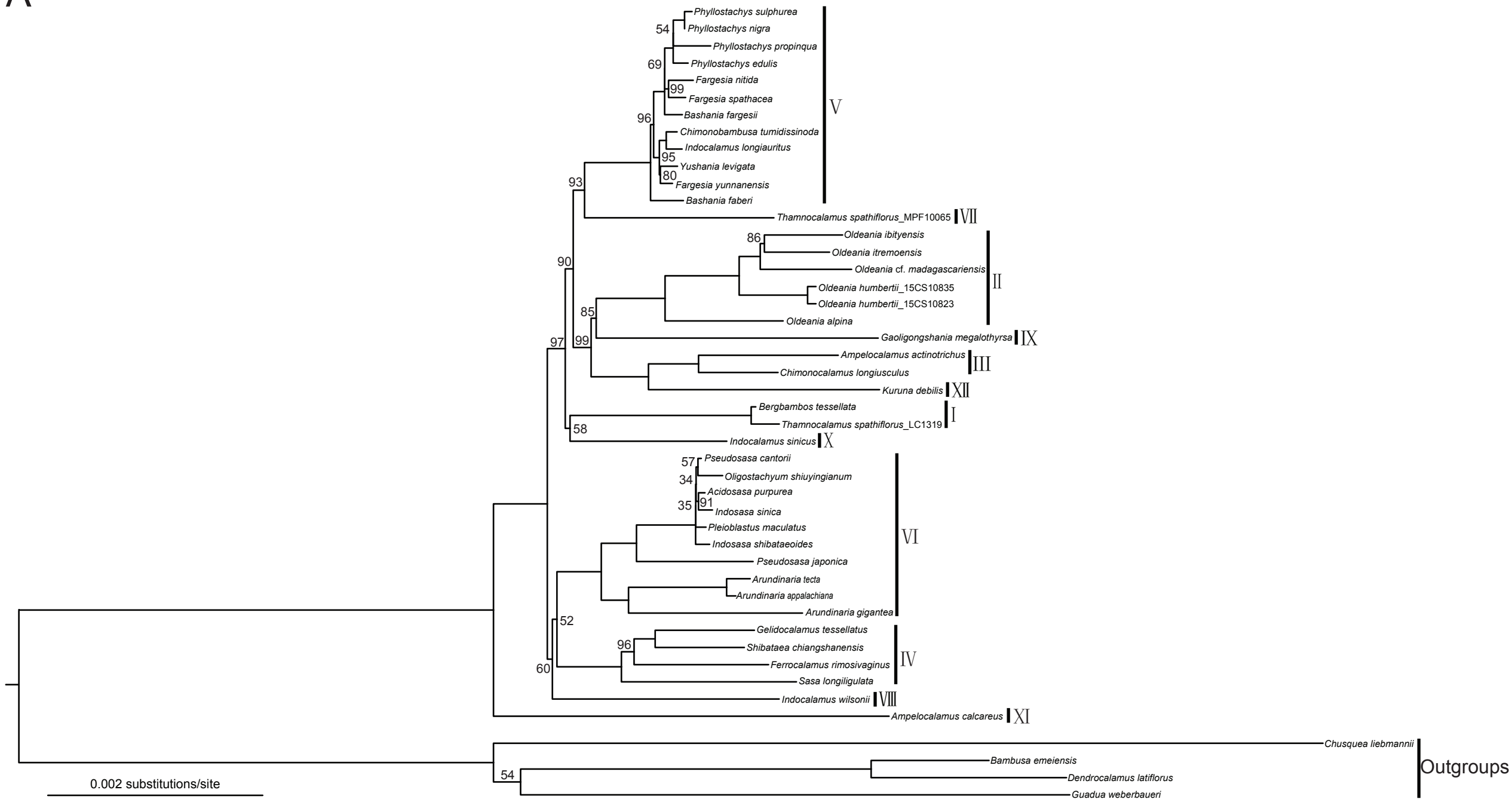

B

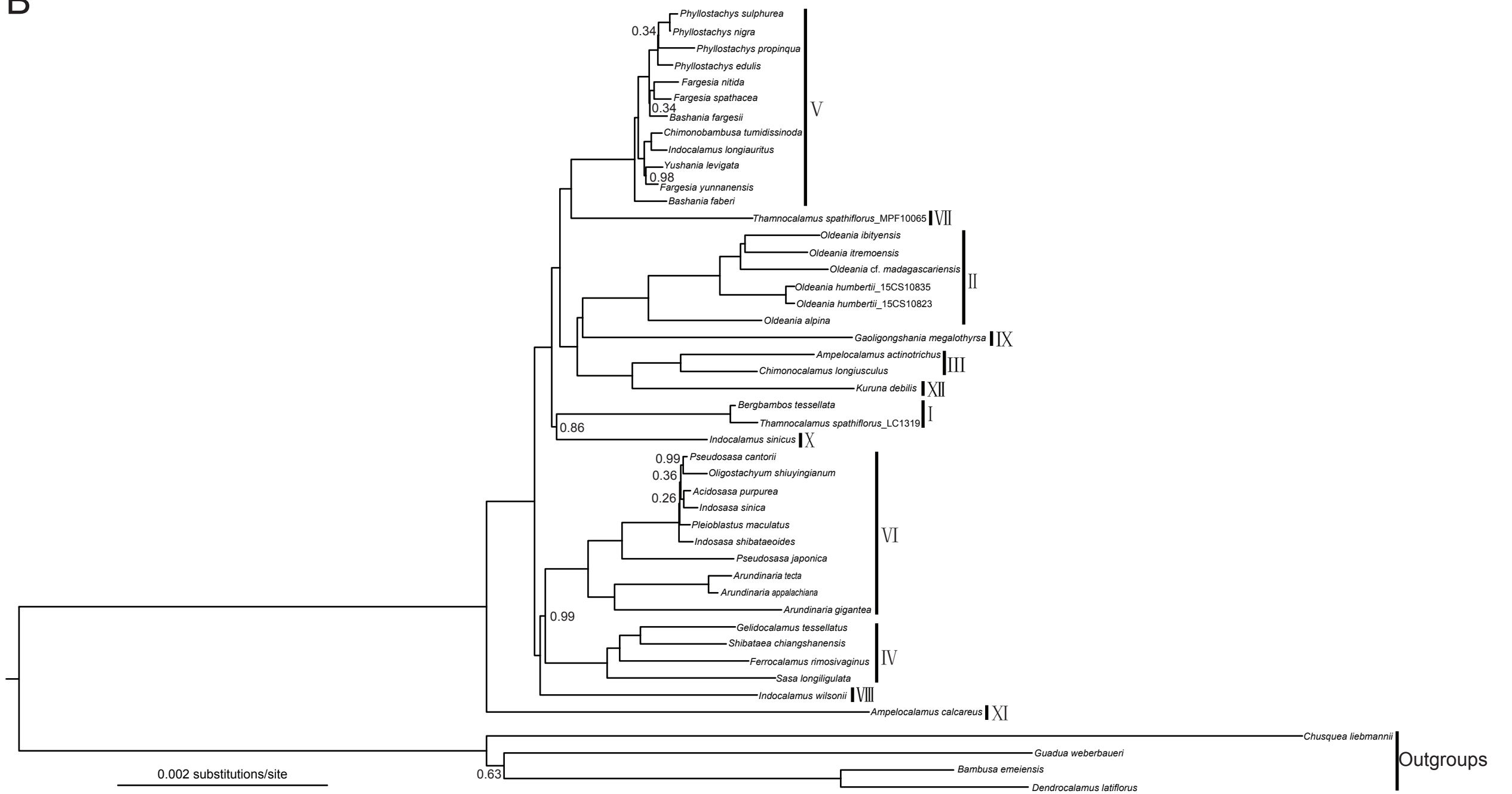

Supplement: Supplementary file 2 — Phylogenetic relationships among major lineages of Arundinarieae based on complete plastid genomes in unpartitioned maximum likelihood (ML) (A) and Bayesian analysis (B). Values associated with the nodes indicate the ML or Bayesian support values and the unlabeled nodes receive 100% ML bootstrap support or 1.0 Bayesian posterior probability. (PDF 389 kb) [file 12870_2017_1199_MOESM2_ESM.pdf]

A

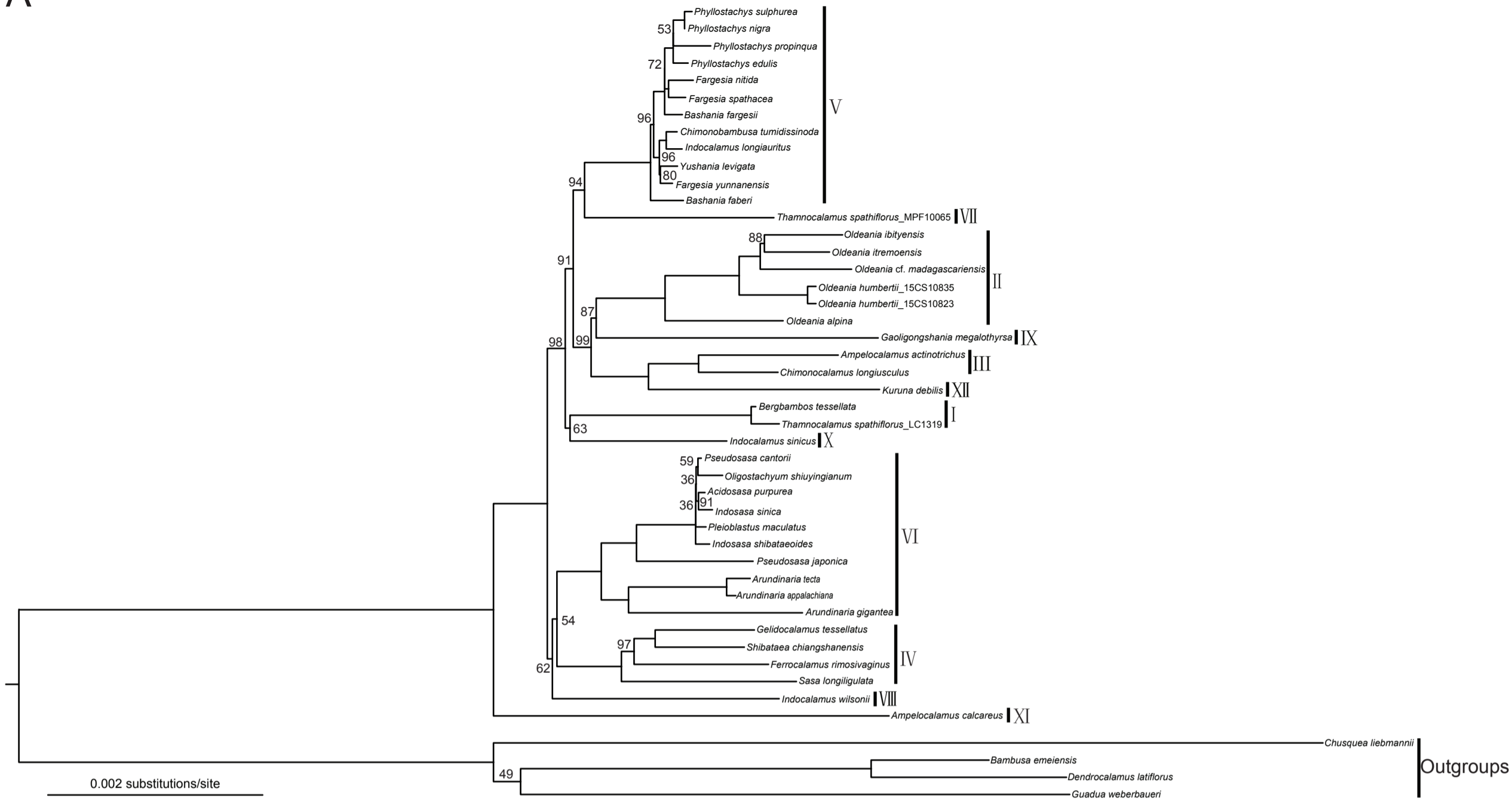

B

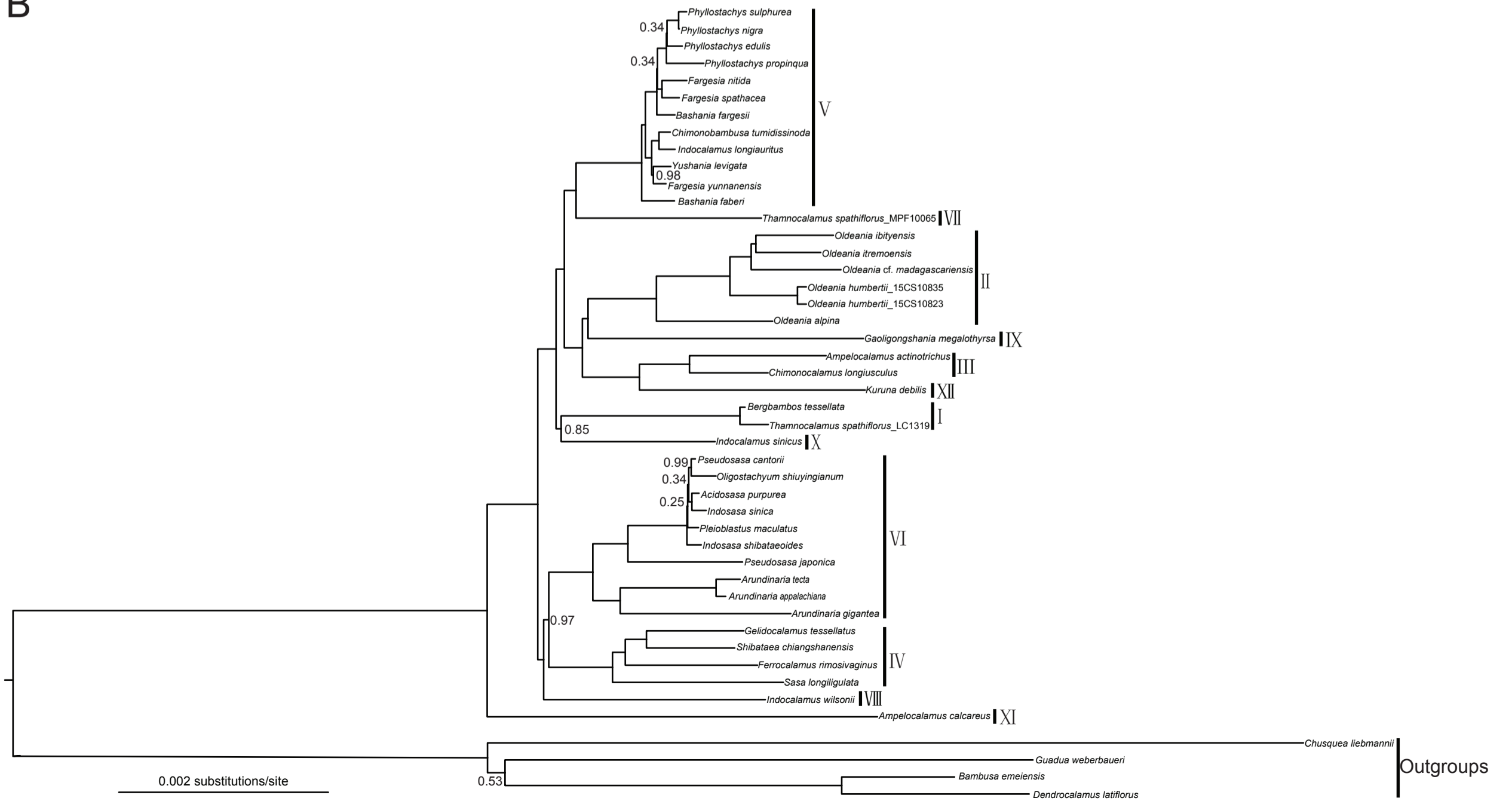

Supplement: Supplementary file 3 — Phylogenetic relationships among major lineages of Arundinarieae based on complete plastid genomes in partitioned maximum likelihood (ML) (A) and Bayesian analysis (B). The partitioned scheme was determined by the software PartitionFinder. Values associated with the nodes indicate the ML or Bayesian support values and the unlabeled nodes receive 100% ML bootstrap support or 1.0 Bayesian posterior probability. (PDF 395 kb) [file 12870_2017_1199_MOESM3_ESM.pdf]

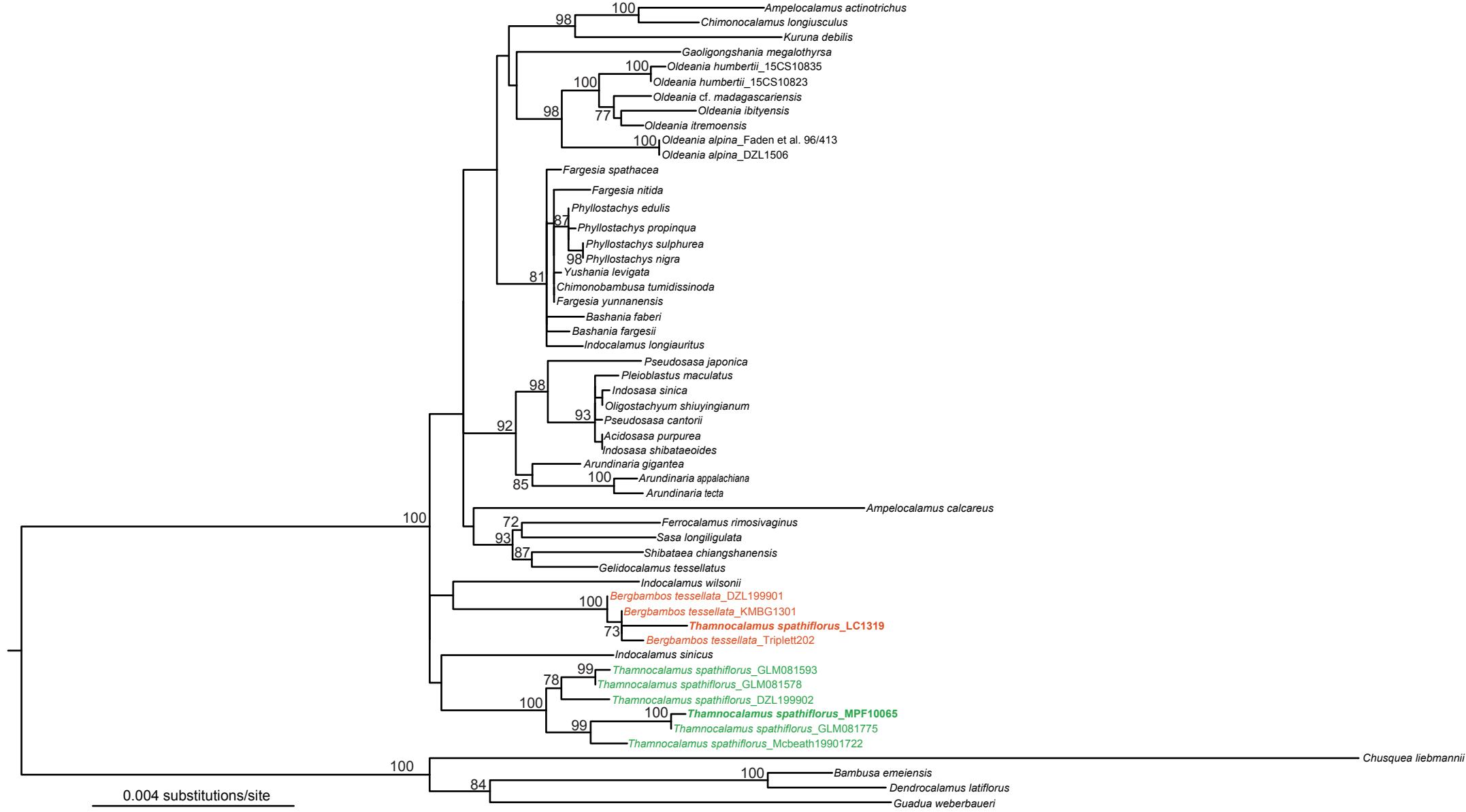

Supplement: Supplementary file 4 — Maximum likelihood (ML) phylogeny of Arundinarieae based on 7 plastid loci (atpI-atpH, psaA-ORF170, rpl32-trnL, rps16-trnQ, trnC-rpoB, trnD-trnT, and trnT-trnL) for the clarification of the phylogenetic placement of Thamnocalamus spathiflorus LC1319. These 7 plastid loci are from Triplett & Clark (2010), Zeng et al. (2010), Wysocki et al. (2015) (references in the main document), and our sampled complete plastid genomes. The DNA sequences of T. spathiflorus LC1319 are from Wysocki et al. (2015) and the T. spathiflorus MPF10056 is our sampling. All the remaining individuals of T. spathiflorus are from Zeng et al. (2010). Values associated with nodes indicate ML bootstrap support values, with only ≥70% shown. (PDF 353 kb) [file 12870_2017_1199_MOESM4_ESM.pdf]

A

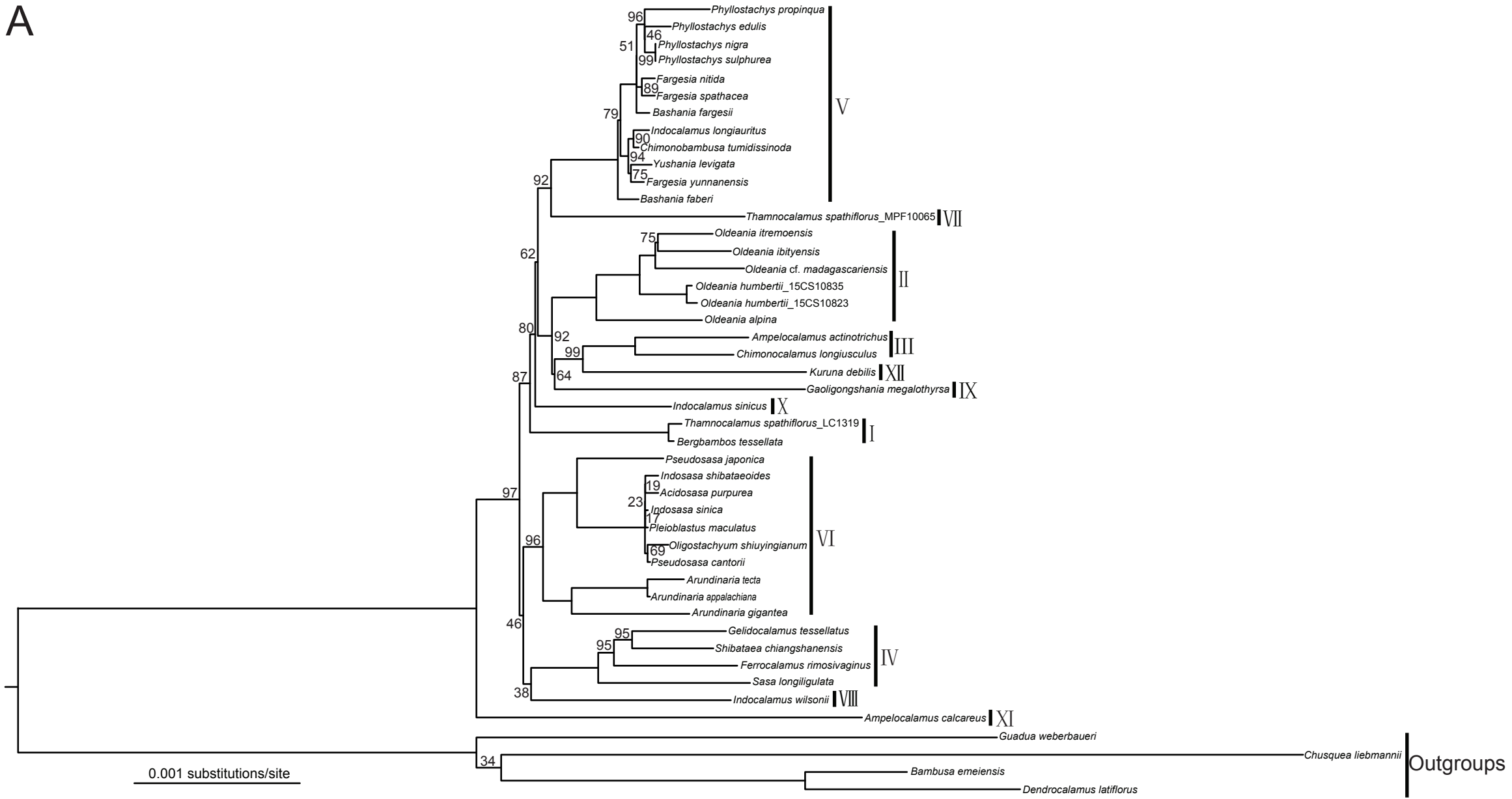

B

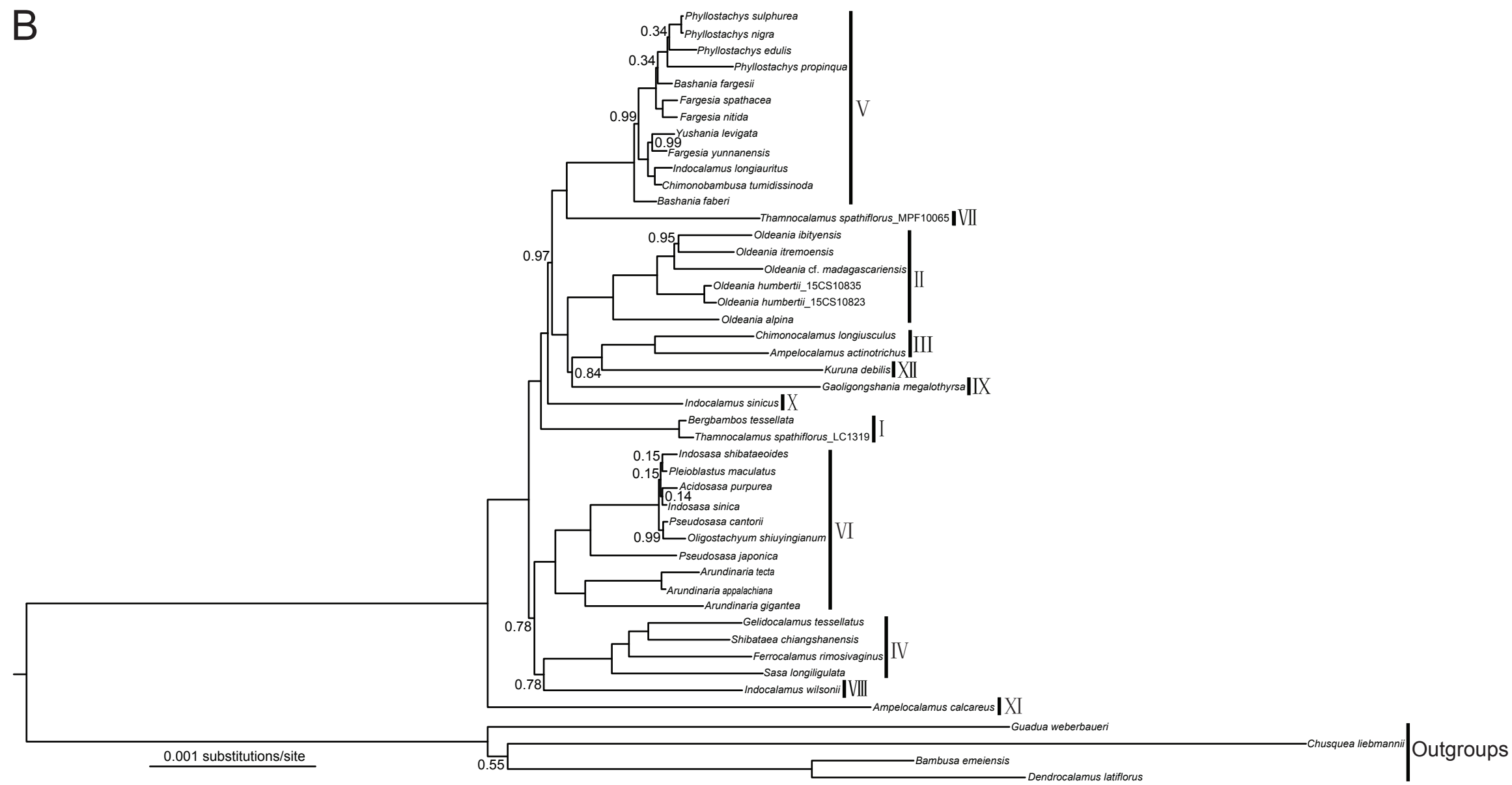

Supplement: Supplementary file 5 — Phylogenetic relationships among major lineages of Arundinarieae based on plastid coding sequences in unpartitioned maximum likelihood (ML) (A) and Bayesian analysis (B). Values associated with the nodes indicate the ML or Bayesian support values and the unlabeled nodes receive 100% ML bootstrap support or 1.0 Bayesian posterior probability. (PDF 409 kb) [file 12870_2017_1199_MOESM5_ESM.pdf]

A

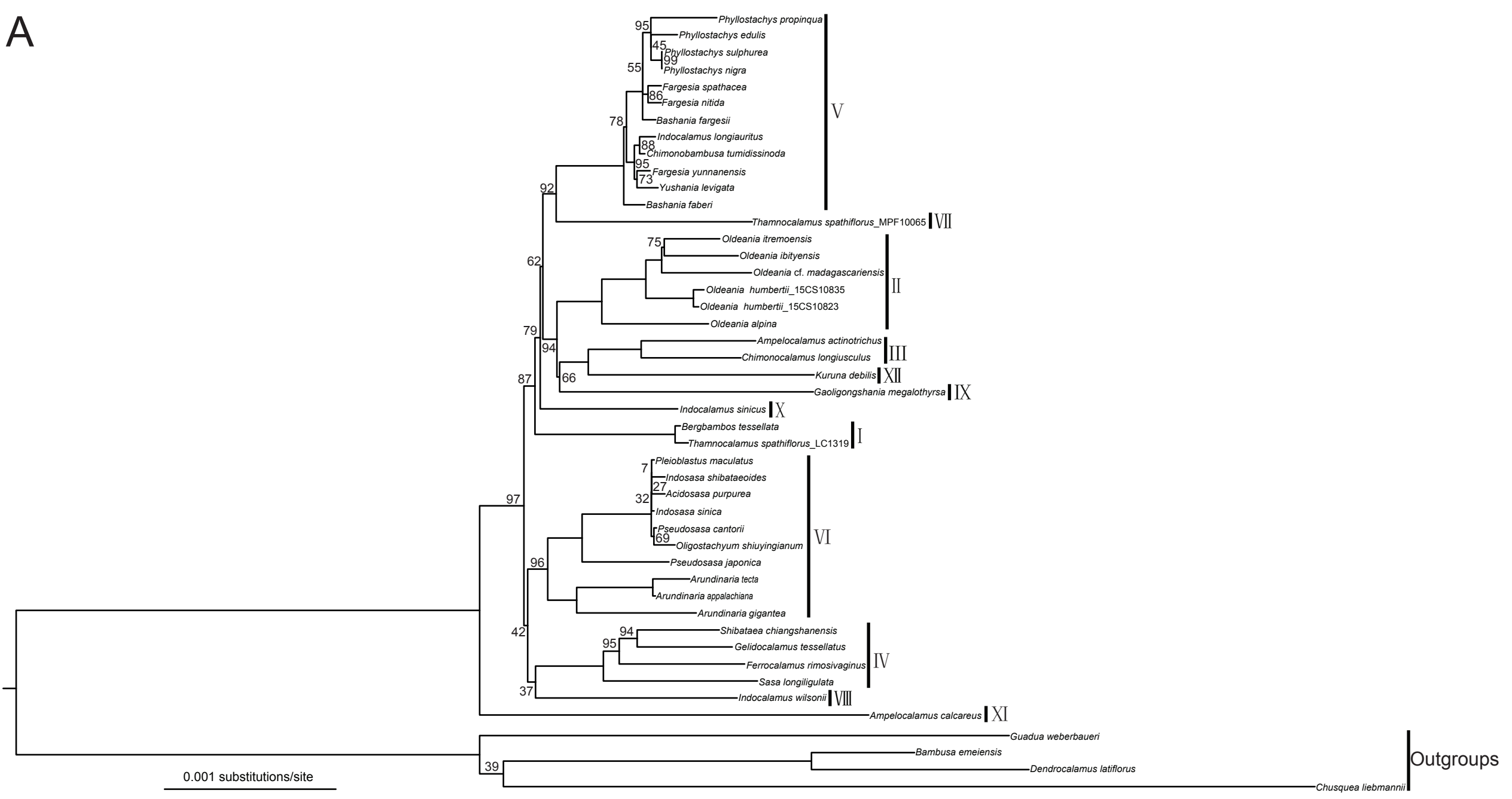

B

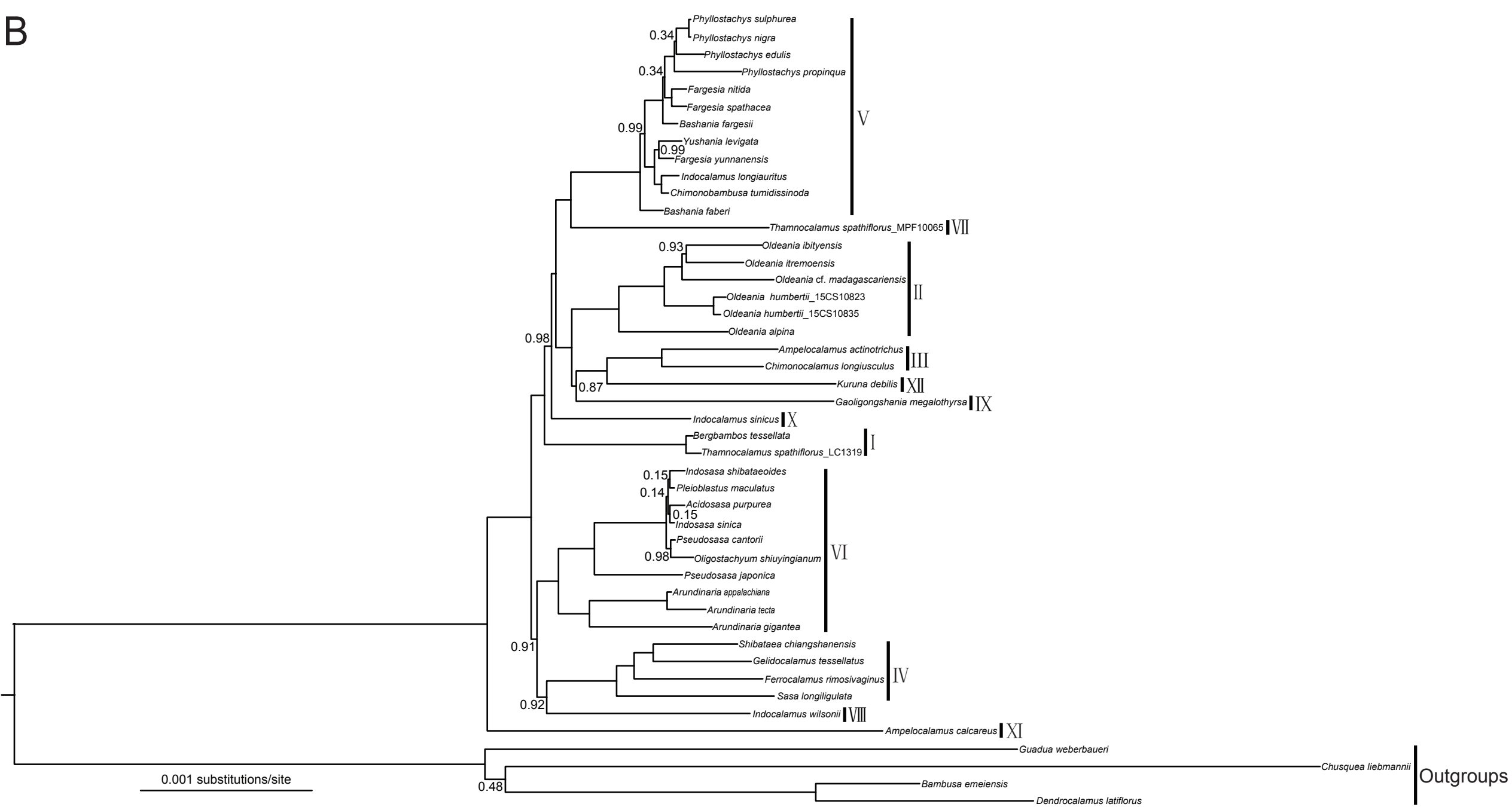

Supplement: Supplementary file 6 — Phylogenetic relationships among major lineages of Arundinarieae based on plastid coding sequences in partitioned maximum likelihood (ML) (A) and Bayesian analysis (B). Five partitions corresponding to the tRNA, rRNA, and three codon positions of protein-coding genes were selected. Values associated with the nodes indicate the ML or Bayesian support values and the unlabeled nodes receive 100% ML bootstrap support or 1.0 Bayesian posterior probability. (PDF 403 kb) [file 12870_2017_1199_MOESM6_ESM.pdf]

A

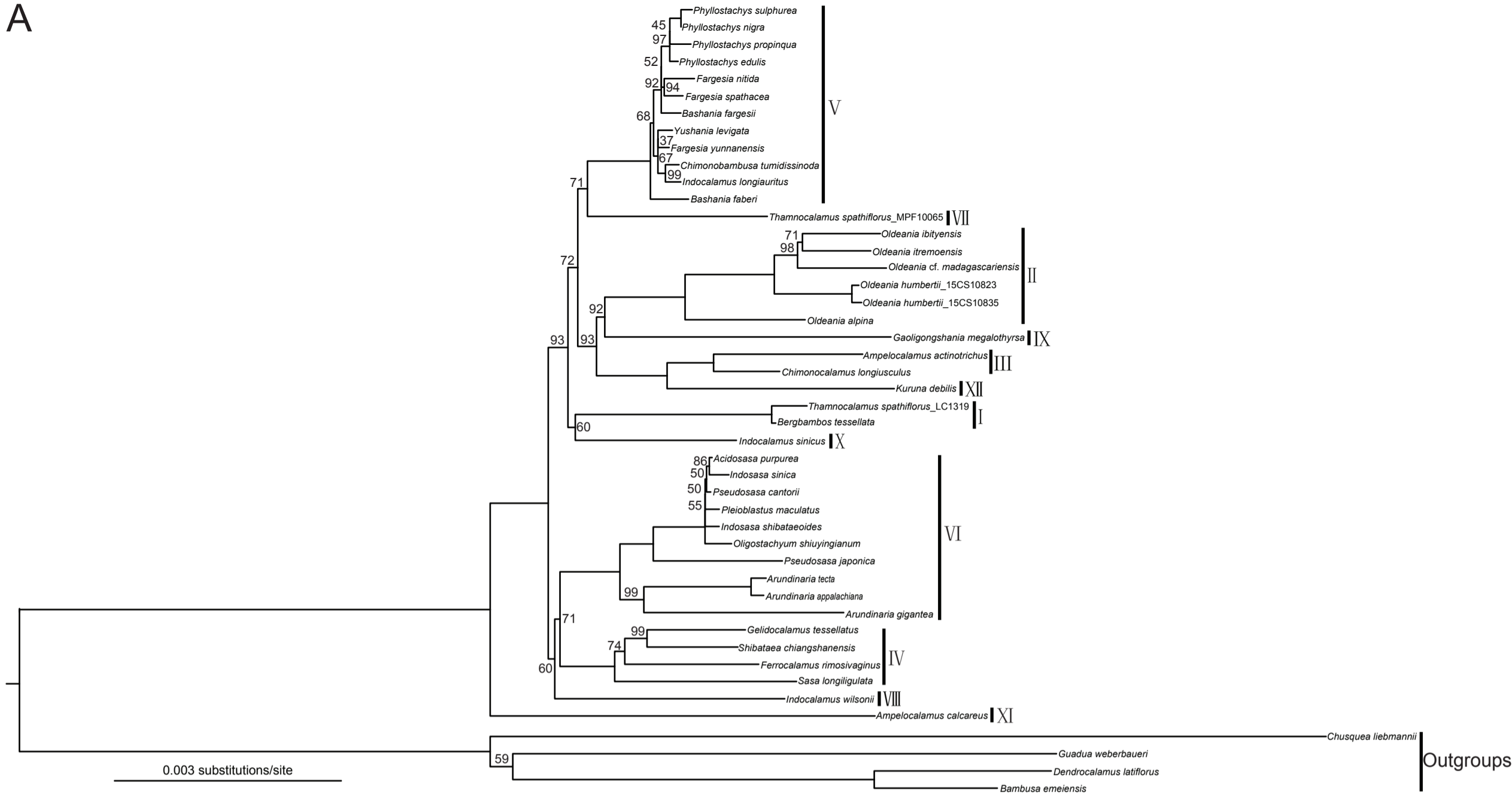

B

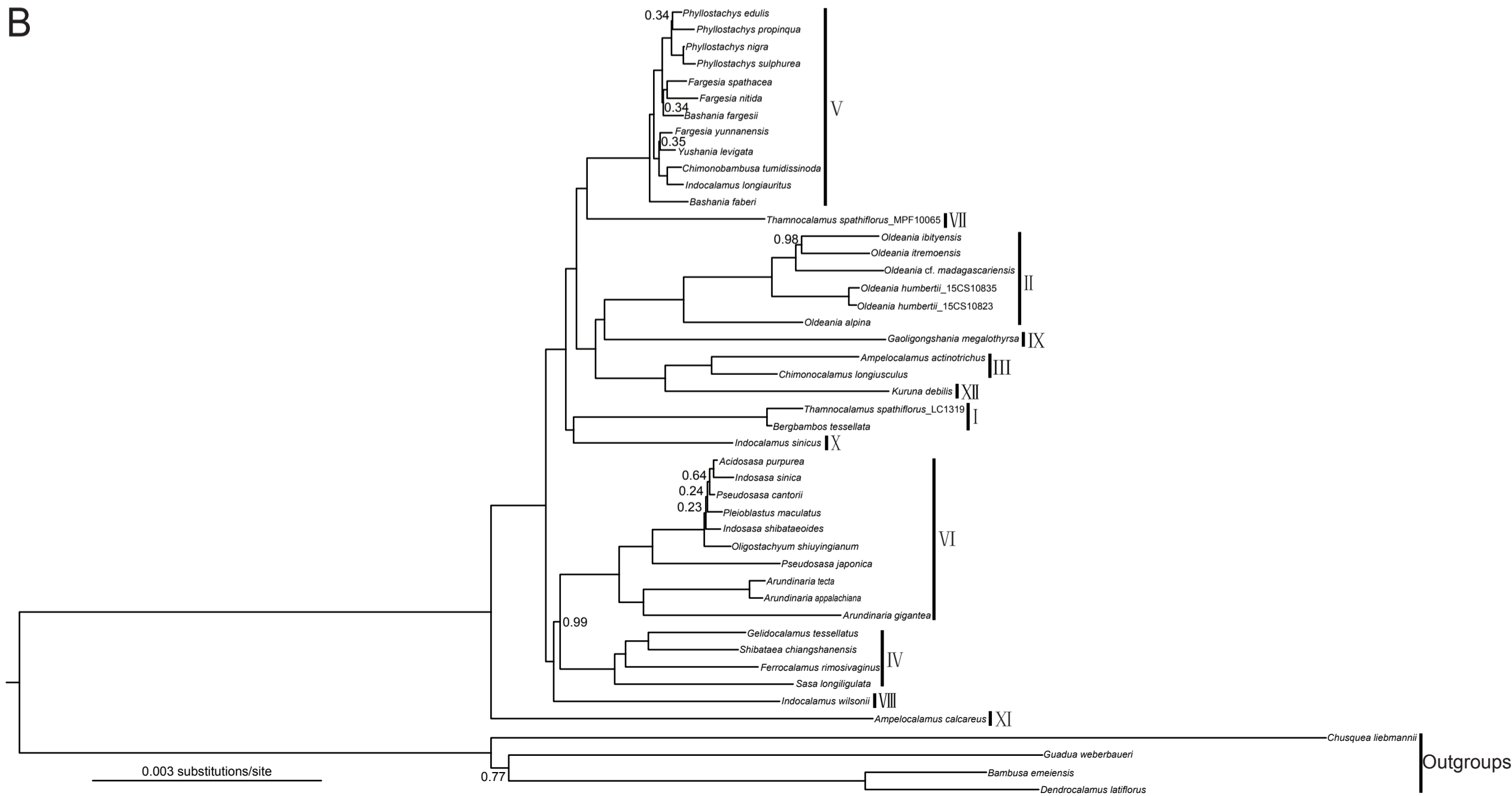

Supplement: Supplementary file 7 — Phylogenetic relationships among major lineages of Arundinarieae based on plastid noncoding sequences in unpartitioned maximum likelihood (ML) (A) and Bayesian analysis (B). Values associated with the nodes indicate the ML or Bayesian support values and the unlabeled nodes receive 100% ML bootstrap support or 1.0 Bayesian posterior probability. (PDF 399 kb) [file 12870_2017_1199_MOESM7_ESM.pdf]

A

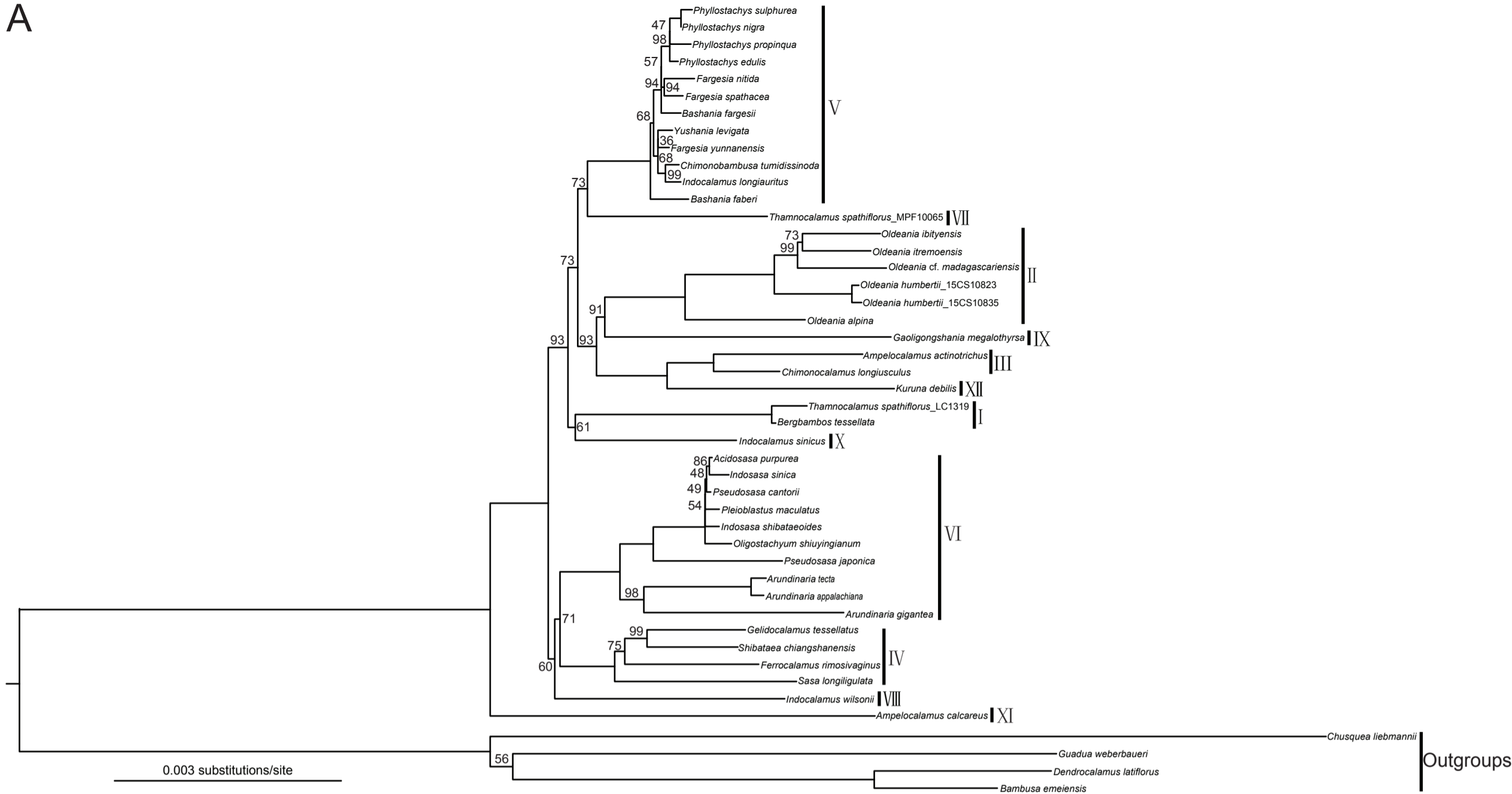

B

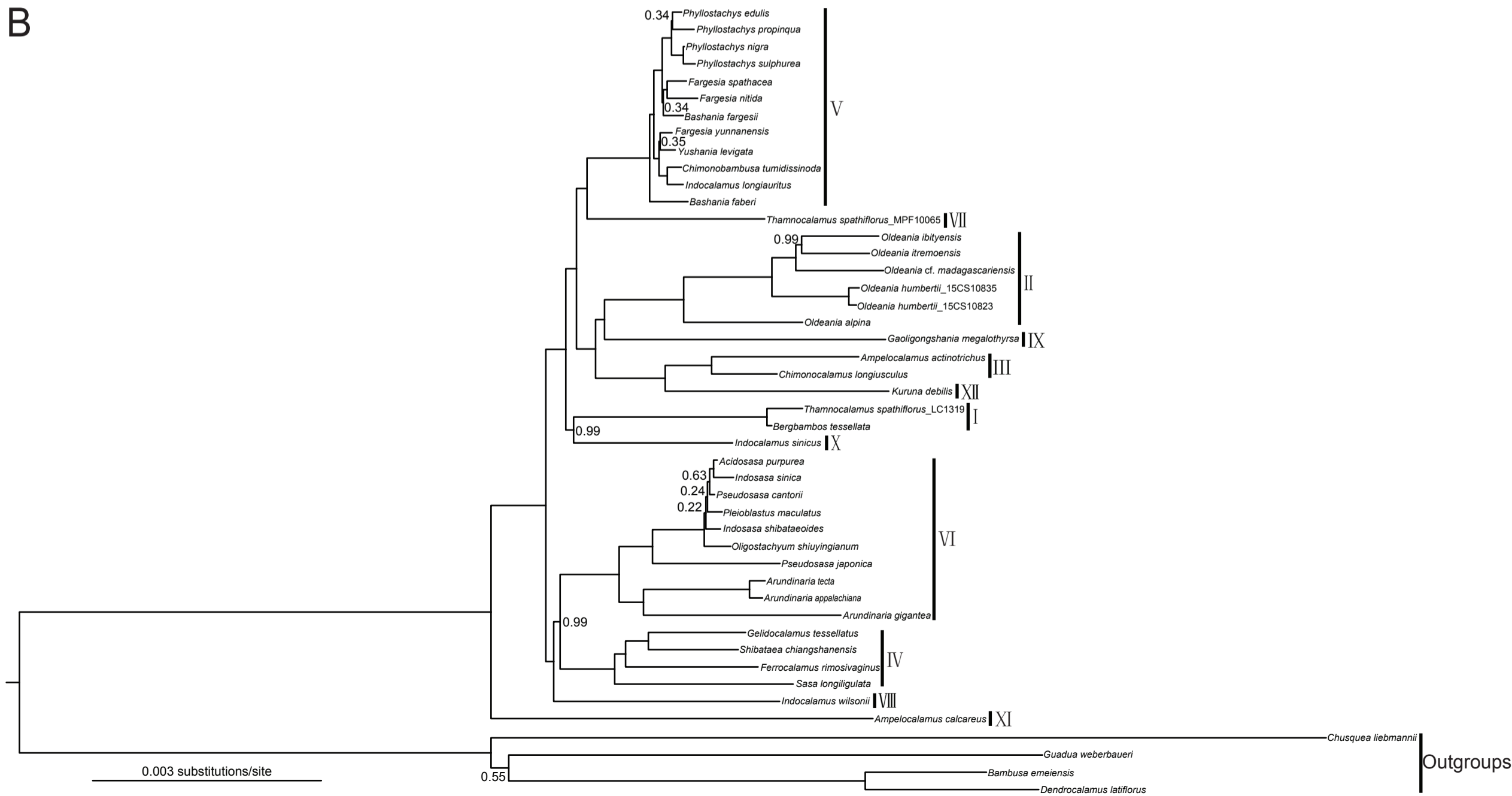

Supplement: Supplementary file 8 — Phylogenetic relationships among major lineages of Arundinarieae based on plastid noncoding sequences in partitioned maximum likelihood (ML) (A) and Bayesian analysis (B). The partitioned scheme was determined by the software PartitionFinder. Values associated with the nodes indicate the ML or Bayesian support values and the unlabeled nodes receive 100% ML bootstrap support or 1.0 Bayesian posterior probability. (PDF 400 kb) [file 12870_2017_1199_MOESM8_ESM.pdf]

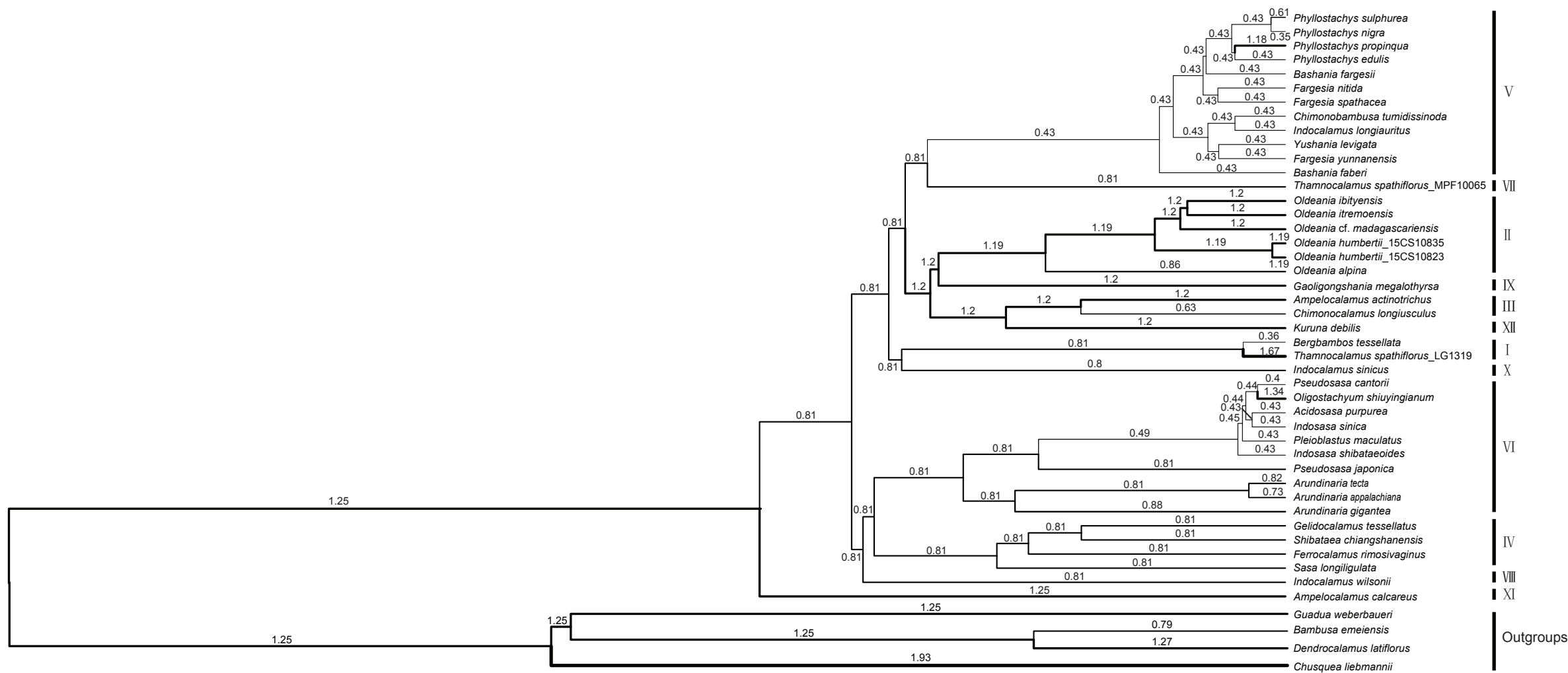

Supplement: Supplementary file 9 — Relative plastid rate of molecular evolution among the lineages of Arundinarieae from Bayesian analysis under the random local clock (RLC) model. Branch thickness is proportional to the inferred median rate for the branch with detail numbers. (PDF 365 kb) [file 12870_2017_1199_MOESM9_ESM.pdf]

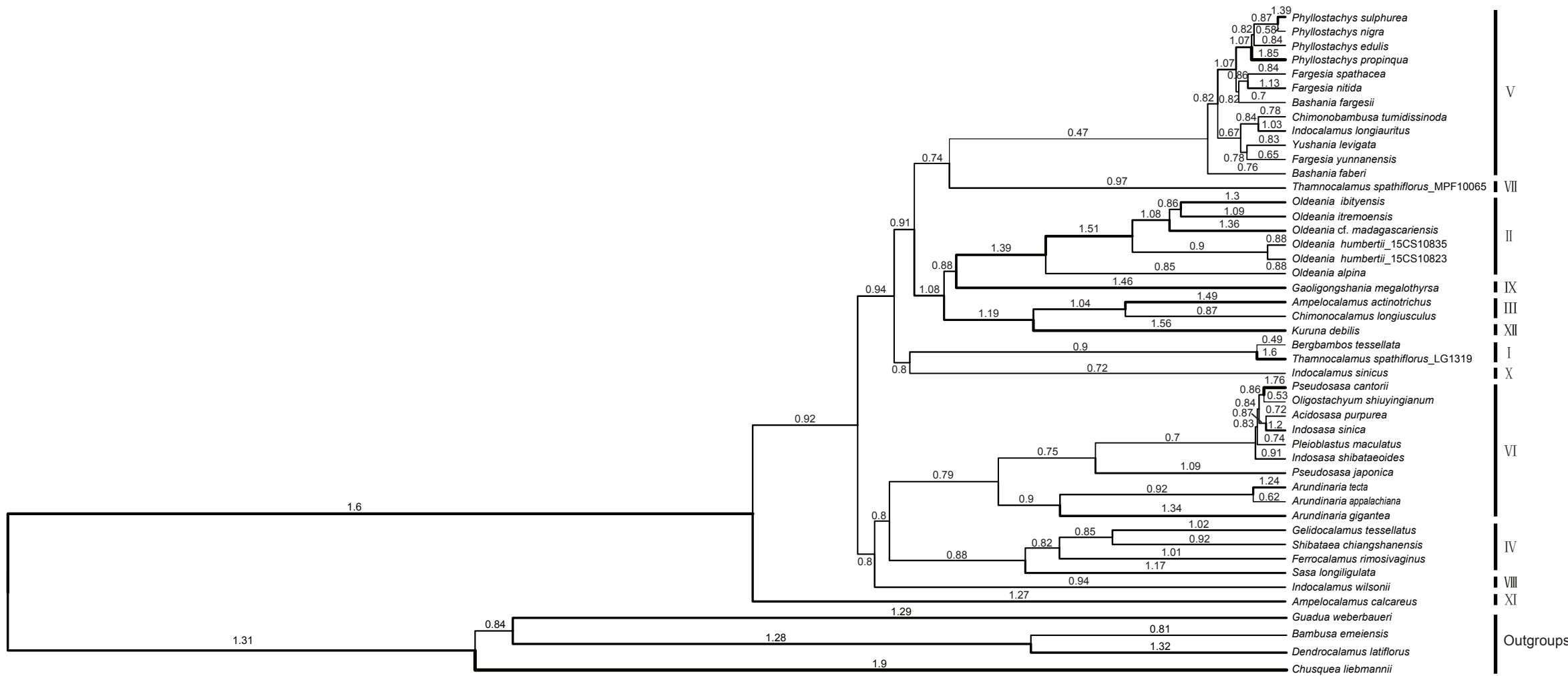

Supplement: Supplementary file 10 — Relative plastid rate of molecular evolution among the lineages of Arundinarieae from Bayesian analysis under the lognormal uncorrelated relaxed clock (LURC) model. Branch thickness is proportional to the inferred median rate for the branch with detail numbers. (PDF 372 kb) [file 12870_2017_1199_MOESM10_ESM.pdf]
