# Supplementary material for: Negative correlation between rates of molecular evolution and flowering cycles in temperate woody bamboos revealed by plastid phylogenomics
Source: BMC Plant Biol. 2017 Dec 21;17:260. doi: 10.1186/s12870-017-1199-8 (PMC5740905; doi:10.1186/s12870-017-1199-8)
Supplement: Supplementary file 11 — Sequence length and model selected for the three data sets used in the study. (DOC 29 kb) [file 12870_2017_1199_MOESM11_ESM.doc]

Table S2. Sequence length and model selected for the three data sets used in the study.

| Data set | Aligned length (bp) | Model |
| --- | --- | --- |
| Complete plastid genomes | 124,679 | TPM1uf + Γ + I |
| Coding sequences | 62,831 | TVM + Γ |
| Noncoding sequences | 61,779 | TPM1uf + Γ + I |
