# Supplementary material for: Negative correlation between rates of molecular evolution and flowering cycles in temperate woody bamboos revealed by plastid phylogenomics
Source: BMC Plant Biol. 2017 Dec 21;17:260. doi: 10.1186/s12870-017-1199-8 (PMC5740905; doi:10.1186/s12870-017-1199-8)
Supplement: Supplementary file 12 — Sequence length and model selected for each data partition of the coding data set. (DOC 31 kb) [file 12870_2017_1199_MOESM12_ESM.doc]

Table S3. Sequence length and model selected for each data partition of the coding data set.

| Partition | Partition identity | Number of sites | Model |
| --- | --- | --- | --- |
| 1 | Codon position 1^st^ combined | 18,609 | TVM + Γ |
| 2 | Codon position 2^nd^ combined | 18,609 | TrN + Γ |
| 3 | Codon position 3^rd^ combined | 18,609 | TVM + Γ + G |
| 4 | tRNAs combined | 2343 | K80 + Γ |
| 5 | rRNAs combined | 4661 | F81 |
